# Biogeochemical properties of blue carbon sediments influence the distribution and monomer composition of bacterial polyhydroxyalkanoates (PHA)

**DOI:** 10.1007/s10533-022-01008-5

**Published:** 2023-01-10

**Authors:** Anthony Grey, Ricardo Costeira, Emmaline Lorenzo, Sean O’Kane, Margaret V. McCaul, Tim McCarthy, Sean F. Jordan, Christopher C. R. Allen, Brian P. Kelleher

**Affiliations:** 1 School of Chemical Sciences, Dublin City University, Glasnevin, Dublin 9, Ireland; 2 The School of Biological Sciences, Queen’s University Belfast, Belfast, Northern Ireland; 3 Department of Chemistry, University of Kansas, Lawrence, 66045 USA; 4 National Centre for Geocomputation, Maynooth University, Maynooth, Ireland; 5 Insight SFI Research Centre for Data Analytics, Dublin City University, Dublin 4, Ireland

**Keywords:** Blue carbon, Coastal wetland, Biogeochemical properties, Polyhydroxyalkanoates, PHA, Carbon storage and cycling, Elevation gradient

## Abstract

Coastal wetlands are highly efficient ‘blue carbon’ sinks which contribute to mitigating climate change through the long-term removal of atmospheric CO_2_ and capture of carbon (C). Microorganisms are integral to C sequestration in blue carbon sediments and face a myriad of natural and anthropogenic pressures yet their adaptive responses are poorly understood. One such response in bacteria is the alteration of biomass lipids, specifically through the accumulation of polyhydroxyalkanoates (PHAs) and alteration of membrane phospholipid fatty acids (PLFA). PHAs are highly reduced bacterial storage polymers that increase bacterial fitness in changing environments. In this study, we investigated the distribution of microbial PHA, PLFA profiles, community structure and response to changes in sediment geochemistry along an elevation gradient from intertidal to vegetated supratidal sediments. We found highest PHA accumulation, monomer diversity and expression of lipid stress indices in elevated and vegetated sediments where C, nitrogen (N), PAH and heavy metals increased, and pH was significantly lower. This was accompanied by a reduction in bacterial diversity and a shift to higher abundances of microbial community members favouring complex C degradation. Results presented here describe a connection between bacterial PHA accumulation, membrane lipid adaptation, microbial community composition and polluted C rich sediments.

**Graphical Abstract:**

Geochemical, microbiological and polyhydroxyalkanoate (PHA) gradient in a blue carbon zone.

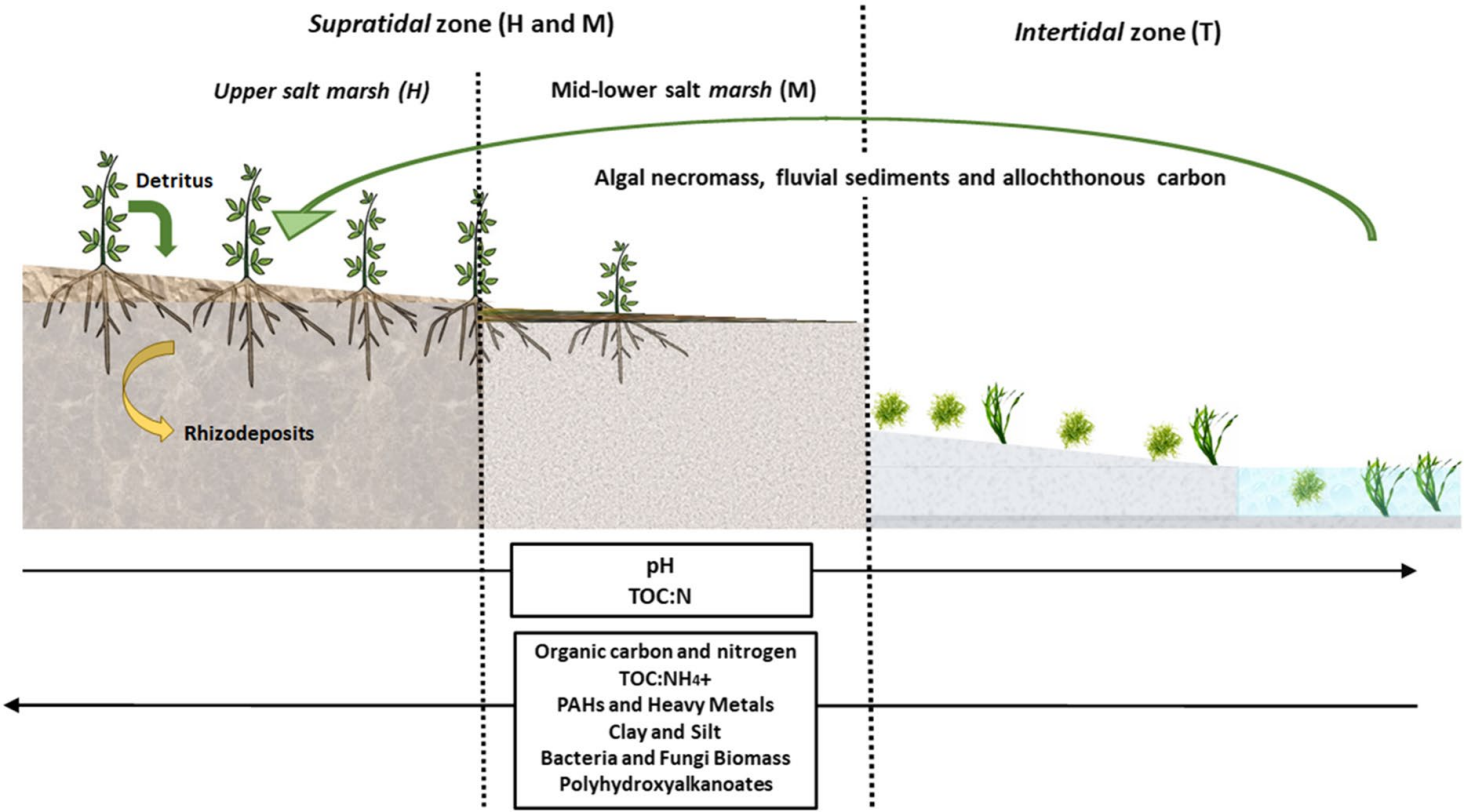

**Supplementary Information:**

The online version contains supplementary material available at 10.1007/s10533-022-01008-5.

## Introduction

Coastal wetlands are some of the world’s most important blue carbon sinks, estimated to capture between 235 and 450 Tg C every year. The rhizosphere synergy of plant-sediment-microbial interactions is a primary driver of C accumulation in water-logged vegetated coastal ecosystems where fluctuating redox conditions and potentially high anthropogenic impacts present dynamic metabolic challenges within the rhizosphere horizons (Ortíz-Castro et al. 2009; Jacoby et al. 2017; Barré et al. 2018). Coastal wetland sediments provide a blueprint for constructed wetlands by operating as coastal filters through capture, immobilisation and detoxification of heavy metals (e.g. lead and zinc) (Stumpner et al., October 2018), organic pollutants (e.g. Polyaromatic Hydrocarbons (PAH))(Cottin and Merlin 2008) and excessive nutrients (nitrogen and phosphorus) (Vymazal 2007), especially in vegetated coastal ecosystems (VCE). Microbial processing is central to initiating transformations of deposited materials through all stages of wetland development, including a direct contribution to sediment carbon through living biomass and necromass—constituting as much as 50% of the SOM pool (Simpson et al. 2007). Micro-organism exposure to accumulated sediment toxins, strong redox fluctuations, salinity stress and inter-species competition are just some of the challenges associated with biogeochemical cycling in blue carbon sediments (Grandy and Neff 2008; Lv et al. 2016; Horton et al. 2019). Bacteria are known to adapt in response to geochemical gradients through expression of physiological changes including alterations in membrane lipid composition to alter fluidity, accumulation of solutes for osmotic balances, storage of polyphosphates and storage of carbon through accumulation of polyhydroxyalkanoates (PHA) (Ibekwe and Kennedy 1998; Villanueva et al. 2007; Ochoa-Valenzuela et al. 2009).

Polyhydroxyalkanoates (PHAs) comprise a family of polyester compounds intracellularly produced by many gram (−) and gram (+) microbial species for storage of carbon, energy and as a source of reducing-power in microbes (Anderson and Dawes 1990; Lee 1996; Madison and Huisman 1999; Jendrossek 2005). The polymers exist as both homo-polymer and co-polymer that are defined by the chain length and the diversity of monomers present in the biopolymer (Haywood et al. 1991). Generally, PHAs can be divided into three groups, determined by the carbon chain length of the monomers:–(1) short chain length (SCL) PHAs including 3 to 5 carbons, (2) medium chain length (MCL) PHAs including 6 to 14 carbons and (3) the less reported long chain length (LCL) where the number of carbons in the monomer backbone exceed 14 (Goh and Tan 2012; Sagong et al. 2018). Polymer structure is dependent on the substrate available and the metabolic pathway through which the carbon is processed, subsequently a function of the genetic expression of different microbial species (Tsuge 2002; Aldor and Keasling 2003). The presence of PHAs in the environment with numerous types of monomers has been reported by earlier studies (Wallen and Rohwedder 1974; Findlay and White 1983; Findlay et al. 1990). PHAs are regarded as a bioplastic alternative to petrochemical polymers. PHAs are advantageous in this context due to high biodegradability and biocompatibility (Muhammadi et al. 2015), indeed having properties in the range from thermoplastic to elastomers (Koller 2018).

The abundance and structural changes of PHAs in different environments can be used as biomarker evidence of changes within microbial communities as a response to external stress factors (Findlay et al. 1990; Foster et al. 2001; Mizuno et al. 2010; Mizuno et al. 2017). PHA accumulation has been well documented as a cellular response to conditions inhibiting cell division or compromising cell integrity (Ayub et al. 2009; Ong and Sudesh 2016; Sedlacek et al. 2019). The presence of intracellular PHA provides a variety of additional functions when a microorganism faces adverse conditions. The main stresses are reported to be growth-limiting, such as nitrogen, phosphate, sulfur, oxygen or magnesium, dependent on the metabolic needs of the microbe (Anderson and Dawes 1990; Poirier et al. 1995). In this context, numerous experimental reports have demonstrated that the capability for PHA biosynthesis and degradation also substantially enhances the survival of bacteria when exposed to various physical stresses including high temperature (Zhao et al. 2007; Wang et al. 2009), freezing and thawing cycles (Pavez et al. 2009; Obruca et al. 2016), low temperatures (Nowroth et al. 2016), osmotic up-shock (Obruca et al. 2017), oxidative pressure (Kadouri et al. 2003; Koskimäki et al. 2016; Obruca et al. 2016), or exposure to UV irradiation (Slaninova et al. 2018), actively aiding in the upregulation of ATP generation and nucleotide accumulation (Ruiz et al. 2001).

Recent research shows that PHA metabolism plays a critical role in synchronizing cellular carbon metabolism to availability of resources in PHA-producing microorganisms comparing a mutant strain defective in PHA storage vs a wild strain PHA accumulator. When exposed to high C:N conditions, the mutant strain spilled excess carbon through the TCA cycle as liberated CO_2_, while the wild strain redirected assimilated carbon towards the PHA cycle, thus generating biomass (Escapa et al. 2012). Despite many publications implicating the PHA cycle strongly with central carbon metabolism for endowed bacteria (Rothermich et al. 2000; De Eugenio, Escapa, et al., 2010a; De Eugenio, Galán, et al., 2010b; Galán et al. 2011; Arias et al. 2013; Prieto et al. 2016; Blunt et al. 2019), perspectives on environmental PHA distributions and dynamics are limited. PHA metabolism is a trait that has been undoubtedly linked to enhanced bacteria cell survival and proliferation. However, research is sparse on the presence and influence of PHA producers in the biogeochemical dynamics of mixed microbial consortia across marine and terrestrial sediment habitats. Such information may be influential when incorporated into future biogeochemical models accounting for the processes of microbial life and death (Sokol et al. 2022).

**Fig. 1:**
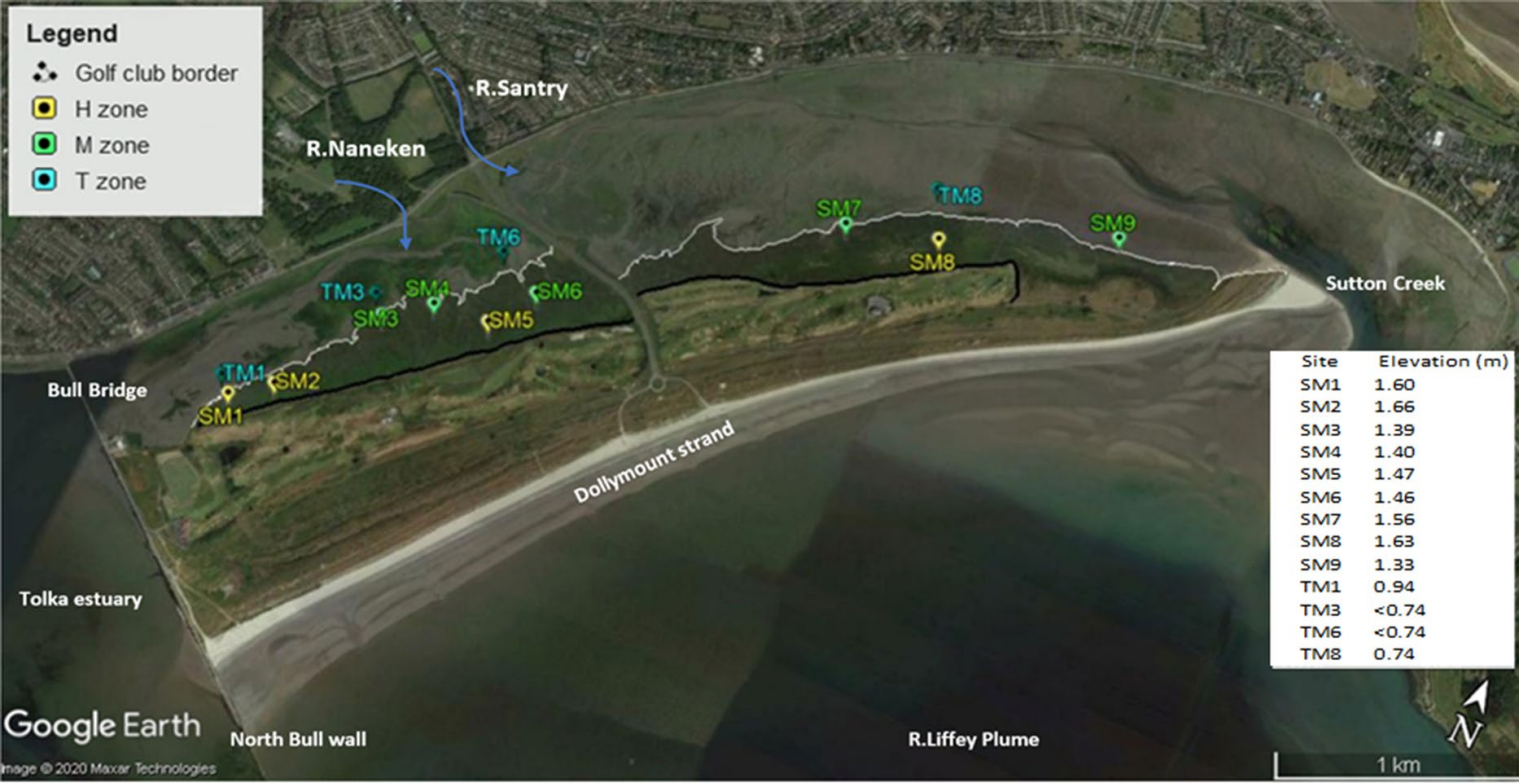
Overview of Bull Island study area and the individual sampling points. Defined sample zones are colour coded as described in the legend: Intertidal zone T = blue, Supratidal mid-Lower marsh zone M = green and Supratidal upper marsh zone H = yellow. Datum elevation of sample sites is reported in meters (m) as calculated using Lidar. Tidal sample points TM3 and TM6 were outside the Lidar survey therefore are reported as less than the minimum tidal elevation point measured i.e. < 0.74 m. (Taken from Grey et al 2022)

To the best of our knowledge this is the first study that focusses on PHA accumulation in coastal wetland sediments in conjunction with the associated sediment geochemistry and microbial community composition. The study was performed along a coastal ecotone encompassing a gradient transect from intertidal sediments (unvegetated and covered daily by tidal water), through vegetated salt marsh sediments (periodically covered by spring tides and flooding events), on Bull Island, Dublin Bay (Grey et al. 2021) (Fig. 1). Previously, results were published from this study where the authors determined the quantity and distributions of bulk geochemical characteristics in sediments through the elevation gradient, including total organic carbon (TOC), total nitrogen (TN), total metals, silt, clay, and also, 16 individual polyromantic hydrocarbon’s (PAH’s) as an indication of anthropogenic input (Grey et al., 2022). Considering the gradient from the Tidal mud zone (T), through the low-mid marsh (M) to the most elevated upper marsh (H), there were significant differences between all zones for many measured environmental variables. The retention of water, metals, PAHs, mud, chloride ions, NH_4_^+^, PO_4_^3−^ and SO_4_^2−^ increased with elevated C concentrations, concurrently where pH significantly decreased. In this current study, we aimed to determine whether microbial community structure and physiological (PHA) response changes spatially in the transition from intertidal to supratidal flats reflecting changes to sediment geochemistry including polycyclic aromatic hydrocarbons (PAH) and metal accumulation along the successional gradient. To assess microbial community composition, we utilised 16 s rRNA metagenomics and PLFA analysis, additionally using signature lipid biomarker (SLB) results to generate indices of bacterial metabolic functioning. Additionally, we hypothesised that PHA concentration and monomer composition diversity would increase with increasing OM, PAH and metals concentration. PHA concentrations and monomer composition were assessed simultaneously with geochemistry, microbial community composition and SLB metabolic indices. This study provides results relating bacterial PHA accumulation to zones of high C sequestration and anthropogenic pollution, highlighting its role as a player in sedimentary microbial C cycling.

## Materials and methods

Detailed description of study site, methodologies and results for all geochemical analysis can be found described by (Grey et al 2022). Methodologies for all parameters in this study can be found in supporting information (SI).

### Sampling

**Fig. 2:**
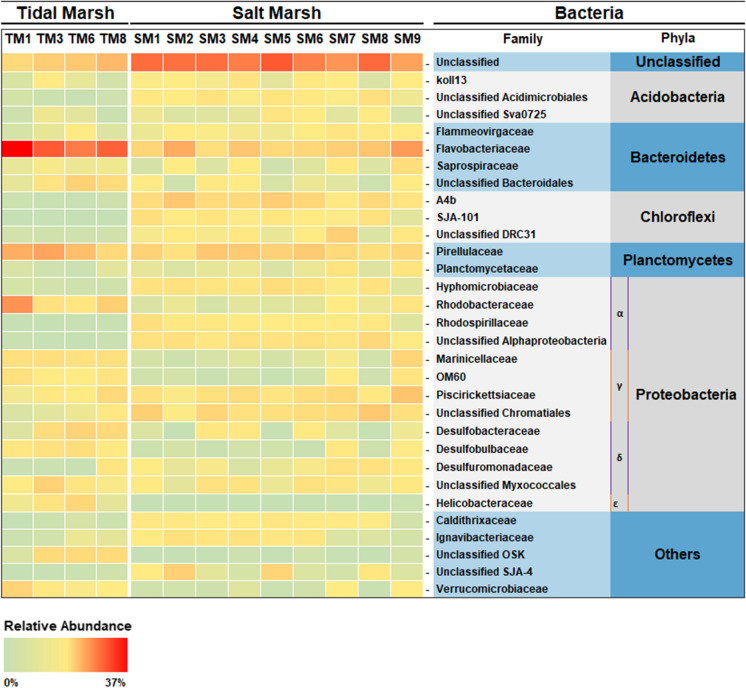
Quantitative taxonomic analysis to family level. Presented are the top 30 abundant bacteria family groups. Percentage relative abundance values can be found in figure SI.7

Sample sites were grouped to represent defined zones across an elevation gradient. The distribution of sites was chosen in order to follow the possible deposition of material transported to sediment areas following tidal inundation. Thus, samples were taken to represent intertidal sediments and supratidal sediments in both upper and mid-lower marsh. Two sample zones on supratidal salt marsh sediments were chosen to further explore an elevation gradient for geochemical variations (Fig. 2).

$${\text{Supratidal}}\left\{ {_{{{\text{Mid}}{-}{\text{lower marsh }}\left( {\text{M}} \right):{\text{SM3}},{\text{ SM4}},{\text{ SM6}},{\text{ SM7}},{\text{ SM9}}}}^{{{\text{High marsh }}\left( {\text{H}} \right):{\text{ SM1}},{\text{ SM2}},{\text{ SM5}},{\text{ SM8}}}} } \right.$$

$${\text{Intertidal }}\left( {\text{T}} \right):{\text{ TM1}},{\text{ TM3}},{\text{ TM6 and TM8}}$$

At each sample site (e.g. SM1), a block of sediment (54,000 cm^3^) was removed intact from the ground. Samples were immediately wrapped in furnaced aluminium foil and flash frozen onsite with liquid N_2_, thus minimising time for possible microbial metabolic processes, preserving the in-situ lipid profile i.e. PLFA and PHA concentration/composition. Bacterial PHA concentrations have been shown to reduce rapidly when redox conditions are altered in a disturbed anaerobic sediment (Findlay et al. 1990; Findlay and Dobbs 1993). The box was sealed with an insulated lid and left to stand for 15 min. Samples were bagged and returned to the lab for storage at − 80 °C prior to subsampling. For geochemical and physical parameter analysis three ~ 200 g subsamples were taken from sediment blocks at each site encompassing a depth range from 0 to 10 cm. Each subsample was taken at separate locations from the block, thereafter treated as individual samples (e.g. SM1 a, b and c) with analytical replications (duplicate or triplicate) performed in respective analysis for each a, b and c samples. This method was applied to all sample sites to account for heterogeneity of these sediment types (Bowen et al. 2009). For microbial lipid extractions and 16 s RNA sequencing, subsamples were taken close to outer edge of the flash frozen sediment blocks and wrapped in furnaced tinfoil and sterile DNA free tubes respectively (Saleh-Lakha et al. 2011; Franchini and Zeyer 2012) (see SI Sects. 4 – 4.2 for description of methodology).

### Lipid biomarker analysis

Freeze-dried sediment aliquots of 5 g were extracted using a modified Bligh and Dyer extraction (Bligh and Dyer 1959; Fang and Findlay 1996; White et al. 1997). Lipids were fractionated using solid phase extraction (SPE) on *Agilent Bond Elute NH*_2_ columns (amino-propyl solid phase − 500 g 3 ml) as described by Pinkart et al. (Pinkart et al. 1998). PLFAs were collected in the polar fraction and derivatised to produce fatty acid methyl esters (FAMEs) utilising sodium methoxide for base catalysed esterification. Mono-unsaturated bonds were identified in subsequent FAMEs by DMDS substitution. The chloroform/acetone fraction was collected for PHA analysis and derivatised using acidified ethanol (at 100 °C for 4 h) to produce ethylated hydroxy acid monomers as described by Findlay et al. (Findlay and White 1983).

## PHA derivatisations and GC–MS analysis

The chloroform/acetone fraction collected for PHA analysis was dried down under a stream of nitrogen. The remaining extract with the polymer dried to the walls of the vial was carefully washed with 3 × 1 ml aliquots of ethanol, followed by 3 × 1 ml aliquots of diethyl ether to remove excessive neutral lipid and free fatty acid compounds likely to cause chromatographic interference. A new GC method was developed for PHA analysis as adapted from Findlay and White (Findlay and White 1983; Tan et al. 2014).

## GCMS analysis of PHA monomer derivatives

The GC column was a fused silica capillary column (30 m × 0.25 mm i.d.) with a film thickness of0.25 μm (HP-5MS, Agilent). Ultra-high purity helium (BIP-X47Sgrade, Air Products) was used as the carrier gas with a flow rate of 1 mL min^−1^. The sample (1 μl) was injected with a 2:1 split ratio. The GC inlet port temperature was set at 250 °C. An initial oven temperature of 60 °C was held for 1 min, followed by a ramp of 5 °C /min to 280 °C, held for 1 min and finally ramped at 25 °C /min to 310 °C for a 20 min hold time. The total run was 66 min for this PHA method. The data was processed using Chemstation software. A series of custom made 3OHA standards (bacteria source) were obtained from *BIOPLASTECH* (Dr. Kevin O’Connor, University College Dublin) and used to generate a standard curve. The mix contained 3-hydroxy butanoic acid (3OHB), 3-hydroxy-valeric acid (3OHV), 3-hydroxy hexanoic acid (3OHH), 3-hydroxy heptanoic acid (3OHHP), 3-hydroxy octanoic acid (3OHO), 3-hydroxy-decanoic acid (3OHD) and 3-hydroxy-dodecanoic acid (3HDoD). The 3-hydroxyacids were subjected to the same derivatisations as per sediment extract. Individual compounds were identified combining mass spectral library databases (NIST and Wiley), standard spectra interpretation, retention times, specific ion extracted chromatograms and published literature. In previous work (not reported here), a pure culture of pseudomonas (obtained from ATCC®) was grown under aseptic conditions using mono-unsaturated fatty acid substrates to facilitate synthesis of mono-unsaturated PHA monomers. The analysis of the isolated unsaturated monomers by GC–MS provided spectra to enhance identification of unknown monomers otherwise unavailable from online databases. Where no standards or examples of spectra were available for LCL monomers and unsaturated monomers, mass spectra fragmentation patterns and respective diagnostic ions were studied from known compounds to estimate RT and thus predicted fragments for potential monomers e.g. Ion m/z 117 representing bond cleavage at the β carbon and molecular ion mass (where possible) to identify addition of a carbon as the chain length of monomers increases.

## Data interpretation

### PLFA metrics

The quantity of PLFAs were expressed as µg g^−1^ freeze dried dry sediment and additionally explored as µg mg OC^−1^ due to spatial variation in C distribution. Total microbial biomass in respective samples was reported as total PLFAs µg g^−1^ and total PLFA represented groups of bacteria, fungi, diatoms, protozoa and algal biomass. Each of the groups was also represented by specific membrane biomarkers traditionally utilised to provide a broader taxonomic classification of the microbial communities present in environmental samples (see Table 1) and the % relative abundance of each biomarker to total PLFA was used to calculate taxonomic information (Table SI.1) (Vestal and White 1989; Zelles 1997, 1999; Bossio and Scow 1998; Li et al. 2007; Chaudhary et al. 2018).

**Table 1:** Biomarker indices used to investigate microbial community composition and physiological response

Biomarker indices	Putative role	Biomarkers used	Reference
Total B.PLFA	The sum of known bacteria membrane PLFAs signifying living bacteria biomass	i14:0, a14:0, i15:0, a15:0, i16:0, a16:0, i17:0, i18:0, a18:0,10Me16:0, Cy17:0, Cy19:0, 16:1ω7, 18:1ω7 and 10Me17:0	Frostegard et al. (1993); Bossio et al. (2005) Willers, Jansen van Rensburg and Claassens (2015)
ADC	Selected membrane polyunsaturated fatty acid (PUFA) PLFAs associated with living Algal, Diatom and Cyanobacteria biomass generally represent organisms predominant in marine intertidal sediments and microbial mats	16:3ω3, 6, 9, 16:2ω4, 7, 16:1ω9, 18:3ω6, 9, 12, 18:2ω4, 7, 20:5ω3, 6, 9, 12, 15, 20:3ω6, 9, 12, 20:1ω9c, 22:6ω3, 6, 9, 12, 15, 19, 22:5ω7, 10, 13, 16, 19 and 22:4ω3, 6, 9, 12	Vestal and White (1989) Rütters et al. (2002)
Actinomycetes	PLFAs methylated at '10' position from the functional group end represent filamentous bacteria order actinomycetales	10Me16:0 and 10: Me17:0	Frostegard et al. (1993) Chaudhary et al. (2018)
Fungi	Sum of signature PLFAs reported to represent total fungal biomass including saprophytic fungi, arbuscular mycorrhizal (AM) fungi and other fungi groups	18:2ω6, 9 and 16:1 ω 5	Olsson et al. (1995) Olsson (1999) Bååth and Anderson (2003)
Protozoa	Sum signature PLFAs reported used to represent protozoa, a group of eukaryotic heterotrophic scavengers and known bacterivores	20:4ω6, 9, 12, 15 and 20:3ω7, 10, 13	Fierer et al. (2003)
B.PLFA:TOC	The ratio of living bacteria biomass to OC content is used as an indicator of C substrate quality or efficiency of C conversion to bacteria biomass. High value indicates higher C conversion to biomass	See relevant biomarker indice	Allison et al. (2007)
Cy:pMUFA	The higher ratio of Cy FA to respective unsaturated FA precursor indicates relatively higher metabolic stress or stationary phase	(Cy17:0 + Cy:19:0)/ (16:1ω7 + 18:1ω7)	Frostegard et al. (1993) Grogan and Cronan (1997)
PHA	Total PHA (μg/g sdw) is represented by the sum of all quantified hydroxy acid monomer inclusions in relevant lipid fraction	3-hydroxy acid monomers e.g. 3OH4:0, 3OH-5:0, 3OH10, 3OH12:1w, 3HO12:O, 3OH13:O, 3OH14:1w, 3OH14:O, 3OH16:1w, 3OH18:1w and 2Me4OHV	R. H. Findlay and White (1983) Green and Scow (2000); Foster, Saufi and Holden (2001)
PHA:B.PLFA	A PHA to B.PLFA ratio has been reported to indicate elevated levels of bacteria stress or cyclic metabolic process in response to diel fluctuations especially in occurrences of syntrophic mutualism	See relevant biomarker indice	Rothermich et al. (2000) McKinley, Peacock and White (2005)

### PHA metrics

Total PHA was calculated as the sum of individual ethyl esters of 3-hydroxy acid monomers and expressed as PHA µg g^−1^ sediment and additionally explored as PHA µg mg OC^−1^. PHA: B.PLFA ratio was used as a stress index during data interpretation calculated by dividing total PHA µg g^−1^ by total B.PLFA µg g^−1^ (see Table 1).

### Bacteria Metagenomics methodology

16S rRNA amplicon sequencing metagenomics data was acquired through outsourcing of extracts. Due to associated costs, multiple samples at each site was not feasible and one composite sample from each of the 13 sites was analysed. Therefore, 3 portions (~ 5 g) were collected from each sample taken per site for geochemical analysis (e.g. SM1 a, b and c), afterwards screened, combined, and homogenised before a subsample (~ 1 g) was taken for extraction. Total metagenomics DNA from sediment samples was used for amplification and sequencing of bacterial 16S rRNA genes at Molecular Research LP (Mr. DNA, USA). Amplicons of the 16S rRNA gene were generated using primers targeting the V4 variable region (515/806) (Soergel et al. 2012) with a barcode on the forward primer. A 30 cycle PCR reaction was performed using the HotStarTaq Plus Master Mix Kit (Qiagen, USA). Briefly, DNA was denatured at 95 °C for 5 min, amplified with 28 cycles of denaturation at 94 °C for 30 s, annealing at 53 °C for 40 s and extension at 72 °C for 1 min, and finally extended for 5 min at 72 °C. PCR products were purified with calibrated AM Pure XP Beads (Beckman Coulter Inc., USA) and DNA libraries were prepared using an Illumina TruSeq DNA library protocol (Illumina Inc., USA). Sequencing of 2 × 300 bp (PE) amplicon libraries was performed on the Illumina MiSeq System using MiSeq Reagent Kit v3 chemistry (Illumina Inc., USA).

### Shotgun sequencing

Total metagenomics DNA isolated from sediment samples were used for whole metagenome shotgun sequencing at Queens University Belfast (QUB). The Nextera XT DNA Library Prep Kit (Illumina Inc., USA) was used for metagenomics library preparation. DNA libraries of 2 × 150 bp (PE) were sequenced with Illumina HiSeq 2500/HiSeq 4000 Systems using the latest SBS chemistry (Illumina Inc., USA). In total, 13 total metagenomes, and 13 16S rRNA amplicon datasets were generated using Illumina next generation sequencing technologies. Datasets sequenced in this study were identified as SM1, SM2, SM3, SM4, SM5, SM6, SM7, SM8, SM9, TM1, TM3, TM6, and TM8.

### Bioinformatics analysis for bacterial community diversity

16S rRNA gene amplicon read pairs were trimmed (Q25) on both ends and merged at the sequencing facility. Quantitative sequencing analysis was carried out using QIIME 1.9.1 (Caporaso et al. 2010). Sequences were demultiplexed and barcodes were removed. Clustering of sequences into OTUs was performed using open-reference OTU picking based on 97% similarity with USEARCH v6.1.544 (Edgar 2010). Sequence alignment was done with PyNAST 1.0 (Caporaso et al. 2010) and taxonomy assignment was done using the most recent Green genes reference database (August 2013) (DeSantis et al. 2006) with the UCLUST algorithm (Edgar 2010). OTUs were used for estimation of sample diversity. Sample diversity analysis and sample cluster analysis were performed using the vegan v2.5‐2 R package (Oksanen et al. 2018),(DT et al. 2015). Briefly, the richness (R) and evenness (J′) metrics were calculated and the Bray–Curtis dissimilarity method was used to construct the principal coordinates analysis of the samples. Metagenomics data was used to generate bacteria functioning indices as described in Table 2.

**Table 2:** Metagenomics data used to generate bacteria community functioning indices

Group(s)/ratio	16 s rRNA identified taxa used for indices
Gram( +) (Monoderm)	Firmicutes, Actinobacteria
Gram(-) (Diderm)	Acidobacteria, Bacteroidetes, Caldithrix, Chlorobi, Cyanobacteria, Fibrobacteres, Gemmatimonadetes, GN02, GN04, OD1, OP8, Planctomycetes, Proteobacteria, SAR406, SBR1093, Spirochaetes, Tenericutes, TM6, TM7, Verrucomicrobia
Alphaproteobacteria:Acidobacteria (αPB:AcB)	Alphaproteobacteria: Hyphomicrobiaceae, Rhodobacteracae, Rhodosprillaceae, Unclassified. Alphaproteobacteria
	Acidobacteria: Betaproteobacteria, Neisseriaceae,, Nitrosomandaceae, Rhodocyclaceae, Methylophilaceae, Comamonadaceae, Burkholderiaceae, Unclassified. Betaproteobacteria
Oligotrophicbacteria:Copiotrophic (OligoT:CopioT)	Oligotrophic: Acidobacteria and Verrucomicrobia
	Copiotrophic: Actinobacteria, Betaproteobacteria, and Firmicutes

### Data processing

Principal component analysis (PCA) and spearman’s rank correlations were applied implemented in XLSTAT was performed to evaluate relationships between variables and investigate the similarity among the 13 sites from Bull Island’s wetlands based on the data of sediment geochemistry, taxonomic composition at both phylum and family level, and metabolic conditions as expressed through indices generated using microbial PLFA and PHA profiles. PCA aided in the identification of inter-correlating variables, thus helping in the selection of representative predictor variables of many geochemical factors for CCA ordination. Significance testing was carried out between groups T Vs M and M Vs H using the non-parametric Kruskal–Wallis test (p < 0.05), as the study intended to explore a gradient from tidal to upper marsh as a consequence of the distance from seawater inundation. Testing was applied to all geochemical variables, 16 s rRNA data, microbial PLFA taxonomic groups, total PHA µg g^−1^. Using metagenomics data, specific bacteria phyla and classes were compiled into taxonomic groups related to community functioning processes in sediments and these groups were utilised in tandem with SLB metrics during CCA to further investigate community response to geochemical changes (see SI Sect. 5 and Table SI.2). Canonical Correspondence Analysis (CCA) was applied to further explore the correlation of the microbial species and environmental variables in each sample site/group. CCA extracts synthetic gradients from the biotic and environmental matrices, which are quantitatively represented by arrows in graphical biplots (ter Braak and Verdonschot 1995). The CCA plots represent overlap of species in relation to a given combination of environmental variables in the studied season. Arrows represent environmental variables, with the maximal influence value for each variable located at the tip of the arrow. Multicolinearity analysis was utilized to select a number of variables with a minimum VIF (< 5) by removing one of the correlated factors where r > 0.700. Thereafter, significant correlations from Spearman’s analysis were used in the discussion to relate variables not included CCA (e.g. PAH, TOC, N and Pb all correlate where r > 0.700). The analysis was carried out using the XLSTAT program for Excel.

## Results

### Bacterial taxonomic classification and distribution

From average reads of 26,344, a total of 56 bacteria phyla (including candidate phyla), 480 families and 622 genera (including unclassified type for the two latter levels) were identified in 16S rRNA bacterial clone libraries. Unknown/unclassified bacterial amplicons represented 3.9–23.5% of counts. In the context of discussing microbial communities in conjunction with SLB analysis, the focus of results is on the broader taxonomic classifications of bacteria phylum and family (Fig. 2). Calculation of richness (number of different species in a community) and evenness (relative abundance of species) were used collectively to examine bacterial diversity. For both richness and evenness, zone M had significantly (p < 0.05) higher values than other groups H and T (Table SI.4 and Figure SI.4). Results for all sample sites combined indicate that the Proteobacteria are the dominant phyla with a mean % relative abundance of 29.00%, followed by Bacteroidetes (17.72%), Chloroflexi (10.07%), Plantomycetes (8.46%), Acidobacteria (4.36%), Actinobacteria (3.75%) and Verrucomicrobia (2.08%) (Table SI.3). Between groups, Proteobacteria showed the highest % relative abundance across marsh zones (H = 26.51%, M = 30.89%), but second highest in the T zone at 29.11%, with no significance in difference between groups (p > 0.05). Within the Proteobacteria phylum, the class alphaproteobacteria (α- Proteobacteria) displayed variation in abundance, with significant preference (p < 0.05) for the H zone (H = 13.14%, M = 9.15%,

T = 7.35%). Gammaproteobacteria (Ɣ) were highest in the mid marsh zone M at 13.20% (H = 8.39%, T = 10.08%), Epsilonproteobacteria (ɛ) and Deltaproteobacteria (δ) showed significant preference (P < 0.05) for the more marine zone T with 2.07% and 9.65% respectively ((ɛ):H = 0.010%, M = 0.064%, (δ): H = 4.77%, M = 8.23%) (Fig. 2).

Also showing significant abundance increase in zone H was bacterium TM6, a recently nominated pathogen due to the lack of diverse metabolic genes, suggesting a reliance on host by-products (Yeoh et al. 2016). In a recent study, bacterivorous flagellate *Spumella elongate* and other protists were shown to be a target species for candidate phyla TM6 bacteria, where cells were infected and lysed (Deeg et al. 2018). Similarly, filamentous, host reliant and DNA recycling (Starr 2018), Saccharibacteria spp (TM7) were highest in zones H. Such interactions may have an impact on the long term structuring of bacteria community composition, especially where distinctive microbes possess capabilities to adapt to change and out-compete more vulnerable species (Matz and Kjelleberg 2005).

## Microbial lipid biomarkers and metabolic indices

### PLFA distribution and 16srRNA indices

Total microbial and total bacteria biomass was highest (p < 0.05) in zone H (56.94 and 28.06 PLFA µg g^−1^), decreasing in zones M (38.27 and 19.67 PLFA µg g^−1^) and T respectively (14.647 and 4.98 PLFA µg g^−1^) (Fig. 5 and Figure SI.1). Tidal zone T had the highest relative abundance of biomarkers indicative of algal, diatom and cyanobacteria (ADC), where concentrations were significantly highest (p < 0.0001). The overall PLFA profile representing predominantly bacteria of gram negative origin. Actinomycetes and fungi were lowest in zone T (0.14 and < 0.01 µg g^−1^) in contrast to increased relative abundance in zones M (3.86 and 0.78 µg g^−1^) and H (4.84 and 2.61 µg g^−1^), where the latter zone had the significantly highest values for each group (p < 0.001). Zone.

**Fig. 3:**
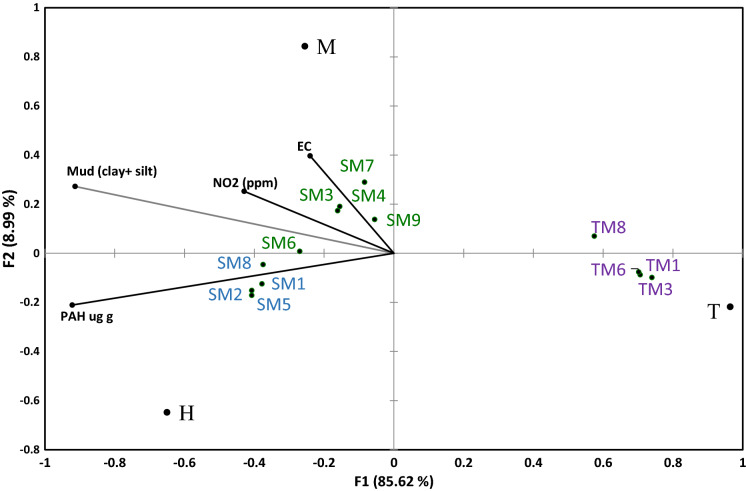
CCA depicting the relationships between representative geochemical variables, microbial community composition and stress status. Graph shows site distributions under the constraints of sediment geochemical variables—% Mud (clay and silt), PAH (µg g), NO_2_^−^ and EC (ms/cm). Further information is supplied in supplementary data where Figure SI.5 displays observations and species distributions at respective zones/sites under the constraints of sediment geochemical variables as generated through CCA

M displayed highest % relative abundance of protozoa (5.01 µg g^−1^) above all zones (H = 3.84 µg g^−1^ and M = 3.87 µg g^−1^), where bacteria diversity (Table SI.4 and Figure SI.4) and NO_2_^−^ were highest of all zones (p < 0.01) (Grey et al. 2022) (Fig. 3).

**Fig. 4:**
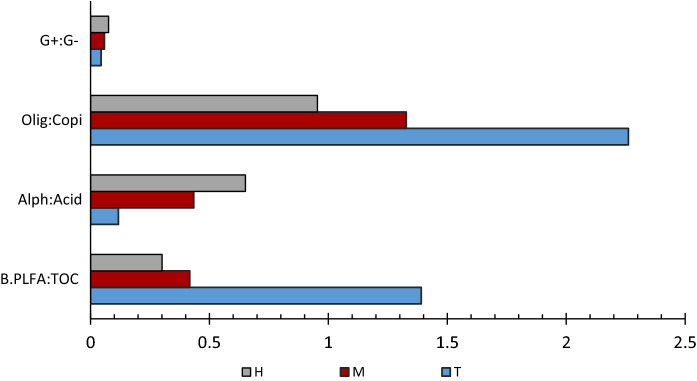
Bar charts comparing ratio indices for shifts in community composition across zones H, M and T. 16 s rRNA sequencing data was used to calculate the following: Oligotrophic: Copiotrophic ratio (Olig:Copi), Alphaproteobacteria: Acidobacteria ratio (Alph:Acid) and Gram + :Gram − (G + :G −). Bacteria PLFA and %TOC were used to calculate the B. PLFA: TOC ratio

Zone T had significantly highest PLFA:TOC and B.PLFA:TOC values (T = 24.63 and 14.46) with both parameters decreasing with distance from the regular tidal zone towards the upper marsh boundary (M = 7.93 and 4.49, H = 6.09 and 3.26) (Fig. 4). Gram negative bacteria stress ratio, Cy:pMUFA and gram positive**:** gram negative ratio values were significantly highest in zone H (0.62 and 0.08), decreasing linearly through zones M (0.41 and 0.06) and T (0.30 and 0.04), demonstrating an opposite trend to ADC biomass measurements (Figure SI.1). The alphaproteobacteria: acidobacteria ratio was highest in saltmarsh sediments showing significant elevation in zone H (H = 0.65, M = 0.51, and T = 0.44), while oligotrophic:copiotrophic ratios were highest in zone T (T = 2.26, M = 1.33 and H = 0.95), both parameters having an opposite trend (Figure SI.1) which is in agreement with previous work by Orwin et al. (Orwin et al*.*, 2018).

### PHA composition and distribution

**Fig. 5:**
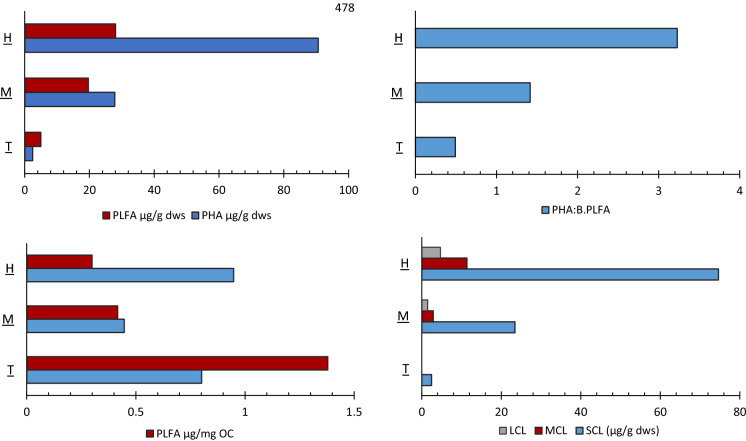
Interpretation of PHA and PLFA distributions at group zones: A) mean PHA and PLFA per µg/g dw sediment, B) mean PHA and PLFA normalised to mg OC, C) mean PHA: PLFA ratios in each zone, D) mean of SCL, MCL and LCL monomers at each sites

Total PHA concentration was significantly highest in zone H (90.63 µg g^−1^) decreasing linearly through zones M (27.82 µg g^−1^) and lowest in zone T (2.44 µg g^−1^) (Fig. 5 and Figure SI.1). The diversity of PHA monomers relating to C chain length were qualitatively similar in groups H and M with a range of monomers from C4 –C18, including saturated and unsaturated (Table SI.4). However, only 2 sites from zone H and 1 site from zone M had all detected monomers SCL, MCL and LCL present (Table SI.2). In zone T, only SCL monomers 3OH4:0 and 3OH5:0 were detected at all sites. The PHA:B.PLFA ratios (Fig. 5) followed the same trend as PLFA stress ratio Cy:pMUFA (Figure SI.1) with significantly highest values (p < 0.05) in zone H (3.50) with a decrease linearly through zone M (1.56), and lowest in zone T (0.46). PHA µg/mg OC in SM samples maintained similar trends (Figure SI.6B), although SM8 was lower than SM4, SM7 and SM9 when compared to PHA ug/g sdw values. In the tidal samples, TM1 had the highest PHA ug/mg OC out of all sample sites, with TM6 and TM8 also showing levels similar to some SM samples (Figure SI.6B).

### CCA: influence of geochemistry on microbial community structure

Results of CCA (Figs. 3 and SI.5) indicate PAH distributions and grain size as major factors influencing microbial community structure across sites with both variables represented on axis 1. NO_2_^−^ and EC influence separation to a lesser degree on axis 2, where both variables measured highest in zone M. The results of the permutation test (p < 0.001) indicate the model is significant and the selected environmental variables or constrained inertia explains 94.6% of variability in microbial ordination. All SM samples were separated from intertidal samples on axis 1, due to significantly lower total PAH and % mud content in zone T.

These differences are also reflected in lower TOC, N, metals, while pH is significantly higher on zone T, as derived from correlation analysis. Zone T bacteria phyla was dominated by Bacteroidetes family Flavobacteriaceae, Plantomycetes and cyanobacteria coinciding with highest ADC biomass, and lowest total bacterial and fungal biomass relative to SM zones. Highest B.PLFA:TOC and oligotrophic:copiotrophic ratios were associated with intertidal sediments, where total PHA and subsequent PHA:B.PLFA ratio were significantly lowest. Proteobacteria appeared to be less influenced by PAH and mud content, instead showing more even abundance across zones. A striking increase in C, N, nutrient and metal accumulation is evident with elevation and a transition to SM sites where PAH and smaller grain size prevail. Associated with higher PAH accumulation, there was a significant increase total bacterial and fungal biomass notable cellulose degraders including Actinobacteria and Fibrobacteres, Chloroflexi, Caldithrix and Saccharibacteria (formerly TM7). This most likely signifies a shift in substrate type and availability in a zone where vegetation became denser and fluvial deposition increases. Concurrently, bacteria stress related indices PHA.B.PLFA and Cy:pMUFA ratios increase, more specifically in upper marsh zone H sites, where the host dependent parasitic bacterium TM6 was significantly highest .

## Discussion

### Geochemical influence on microbial community structure and PHA accumulation

In this study we examined an elevation gradient from unvegetated intertidal through vegetated supratidal sediments (Grey et al., 2022). We aimed to investigate the impact of a geochemical gradient on the structure of microbial community and physiological response as expressed through relative changes in lipid biomarkers, specifically the accumulation of PHA. Many of the bacteria present throughout Bull Island’s lagoon zones have diverse metabolic traits targeting polysaccharide substrates for both aerobic and fermentive assimilation. However, some phyla/families present possess more distinctive niches evident through significant differences in community composition between zones H, M and T, thus revealing a response to geochemical gradients. PHA concentration and monomer structural diversity increased in supratidal vegetated sediments where OC, nitrogen, nutrients, metal and PAH content increased. This coincided with higher levels of bacterial biomass, fungi and complex C degrading bacteria (Actinobacteria, Fibrobacteres and Chloroflexi). Bacterial PHA accumulation and membrane lipid changes are known mechanisms used in sediment habitats to adapt to challenging environments and maintain cellular homeostasis. This is important in the context of C and nutrient cycling processes in vegetated saltmarshes as it enables bacteria to maintain cell integrity and division. Such cellular hardware facilitates the continued role of bacteria in biogeochemical cycling processes in sediments which are subsequently part of a larger mutualistic network including vegetation and sediment microbiology.

### Intertidal sediments

**Fig. 6:**
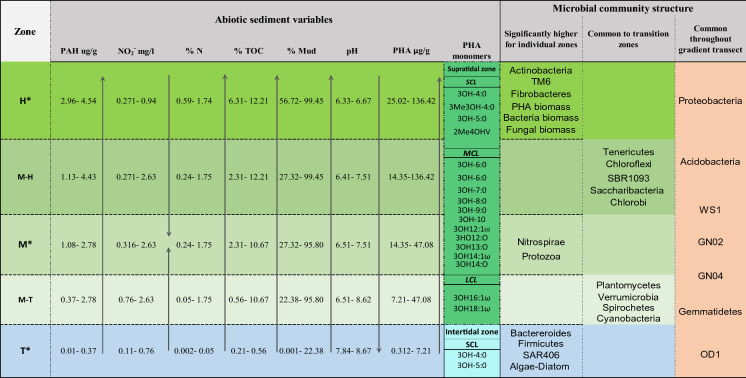
Summary of abiotic variables influencing microbial community structure through a geochemical gradient. Results of correlation analysis (Grey et al., 2022a) displays all other excluded variables which covaried significantly with included abiotic variables above. Gradient zones supratidal salt marsh zones H and M, and intertidal zone T were analysed directly (‘*’) for reported variables. Gradient zones M-H and T-M are simulated or projected zones generated using significant results after Kruskal Wallis testing of metagenomics data and SLB results between defined zones

Total PHA, PHA:B.PLFA and Cy:pMUFA ratios were lowest in zone T (Figure SI.1), where only. SCL 3-hydroxy butanoic and 3-hydroxy valeric acid monomer constituents were detected (Figs. 5 and 6). However, total PHA ug/mg OC was highest in TM1, suggesting a degree of community stress (Figure SI.6). Various species of bacteria from both gram negative and gram positive groups have been shown to utilise glucose, volatile fatty acids (VFAs) and alkanes as a substrate for SCL PHA (Venkateswar Reddy et al. 2017; Mitra et al. 2021). Short chain FAs are plentiful in anaerobic marine sediments, liberated through fermentive processing of sugars and long chain FAs by a range microorganisms including bacteria and some micro algal species (Lovley et al. 1993; Glombitza et al. 2019; Pelikan et al. 2021). These processes described by Burke et al. as ‘dark fermentation’ (Bourke et al. 2017). Previous studies of bacteria community metabolism in marine sediments have demonstrated the use of PHA cycling as a stress response to changes in redox conditions due to temporary sediment disturbance (Findlay et al. 1990). PHA metabolism has also been observed as a diel cycle in marine microbial mat bacteria where interactions between heterotrophs and primary producers displayed synergistic mutualism with temporal changes in redox and substrate availability (Rothermich et al. 2000; Villanueva et al. 2007). Rothermich et al. showed evidence of a distinct diel pattern occurring during PHA metabolism in microbial mats. The authors highlighted how PHA content increased at night and decreased during the day, relating to routine dark energy metabolism and showing little influence from the availability of organic nutrients. Zone T intertidal sediments are subjected fluctuating redox due to tidal regimes a likely process initiating PHA cycling in intertidal bacteria. Although specific PHA accumulating species have not been identified in this study, some known groups of PHA producers were present such as the genus Amaricoccus from the Rhodobacterales order and the sporulating Clostridia class from the fermentive Firmicutes phyla. In BI intertidal sediments, the B.PLFA:TOC and oligotrophic:copiotrophic ratios were highest (Fig. 4), thus suggesting efficient utilisation of OC substrate to bacteria biomass in a community with slower growth rates and lower demand for N (Collins et al. 2016; Kurm et al. 2017). These results are supported by a significantly lower TOC:NH_4_^+^ ratio which indicates high soluble N availability for microbial C assimilation. Zone T sediments had significantly higher pH, a characteristic reported to lower the solubility inhibitory metals in matrices with elevated salinity and anoxic conditions, thus reducing sediment toxicity potential (Riba et al. 2004; Miao et al. 2006).

### Salt Marsh sediments

Total bacteria biomass, PHA and PHA:B.PLFA ratio significantly increased on transition to vegetated supratidal SM sediments, more specifically highest in zone H relative to zone M, showing strongest correlation to Pb concentrations. SM had a greater diversity of PHA monomer constituents which included a range of SCL, MCL and LCL monomers, a characteristic demonstrated by many studies to directly reflect the molecular class of assimilated C substrate (Anderson and Dawes 1990; Steinbüchel et al. 1995; Sharma et al. 2017). In SM sediments elevated C can be attributed to a diversity of sources including vegetation detritus, rhizospheric inputs, fluvial deposits, eukaryotic and prokaryotic biomass. Identification of a variety of MCL PHA monomers through salt marsh zones M and H bear similar structure to the fatty acid structures of identified microbial PLFA constituents. This strongly suggests microbial necromass as a substrate for PHA accumulation through (1) FA uptake from surrounding sediments and, (2) recycling of cell membrane or cytoplasmic lipids. Previous studies focusing on carbon flux dynamics in mixed microbial cultures have shown the incorporation of FAs into PHA granules through the β- Oxidation pathway (Fontaine et al. 2017; Blunt et al. 2018) with availability of a mixture of saturated FAs and long chain unsaturated FAs.

In this study, the Cy: pMUFA bacteria stress ratio was strongly correlated to PHA concentration (p < 0.05), further supporting the occurrence of metabolic stress in SM sediments (Zhila et al. 2015). There was an increase in bacteria associated with more complex C degradation found abundant in zone H, such as Caldithrix and Chloroflexi, the latter characterised by the high abundance of sub-phylum Anaerolineae and families A4b, SJA-101 and UC-DRC31. Another notable increase in vegetated zones M and H occurred with known halophiles, actinobacteria, reflected in a higher gram positive to gram negative bacteria ratio and alphaproteobacteria, both groups with known entophytic members and metabolism favouring high C and N biomass (Rosenberg 2014; Yadav et al. 2018; El-Tarabily et al. 2019). Many studies also show significant PHA accumulating abilities in actinobacteria, across a diversity of challenging environments in response to salinity and metal toxicity (Narancic et al. 2012; Cui et al. 2016). Furthermore, actinobacteria are known to produce allelopathic biochemicals capable of impacting surrounding sediment micro-ecology through excretion of antimicrobial compounds (Patin et al. 2017), which may trigger stress response in other PHA accumulating bacteria. This trait has attracted considerable research from biochemists exploring the medical potential of bioactive metabolites (Manivasagan et al. 2014). Higher OM, N, PAH, metals and increased presence of recalcitrant C degraders coincided with a significantly higher abundance of fungal biomass. Fungi are most often implicated in C degradation of OM and complex biopolymers such as lignocellulose and lignin (D’Annibale et al. 1998; Mäkelä et al. 2015; Cheung et al. 2018). In support of this result, there was a significantly higher abundance of specific cellulose degrading bacteria phylum, Fibrobacteres in the same vegetated salt marsh zones (Ransom-Jones et al. 2012; Rahman et al. 2016). Indeed, some fungi have an apparent tolerance to larger pH ranges (Rousk et al. 2009) and display a high resistance to metal toxicity (Rajapaksha et al. 2004). Furthermore, fungi species are known to produce higher levels of fatty acids than bacteria which aid in the mineralisation of organic P, thus providing soluble P for plants and resident bacteria (B.Sharma et al. 2013). Abundance of protozoa lipids were highest in zone M where metagenomics data showed the highest bacteria diversity. Zone H and T had similar vales for protozoa, despite most strongly contrasting sediment geochemistry, albeit differing in biota composition. The conceptual diagram displayed in Fig. 6 aids in summarising the primary abiotic variables identified as influencing microbial community structure and expression of lipid changes.

### PHA accumulation: a potential player in sedimentary bacteria blue C cycling?

Triggers for bacteria accumulation of PHAs have been studied under a multitude of natural and controlled conditions with primary focus on oxygen and nutrient availability, pH changes, metal toxicity, osmotic pressures and temperature. Albeit, the majority of PHA metabolic studies have been examined in laboratories. In this environmental field study, PHA accumulation was strongly and positively related to OM constituents but also, with Pb, PAH, Cl^−^, pH and the presence of different microbial groups such as fungi, actinomycetes and TM6. Considering the high %N, PO_4_^3−^, SO_4_^2−^ and NH_4_^+^ contents found across salt marsh zones, it would be expected that nutrient availability is not a primary trigger for high PHA accumulation at a broader scale. However, previous studies by Frison et al. have highlighted systematic relationships between PHA biomass, fatty acid uptake and nitrogen removal via NO_2_^−^ in a sequencing batch reactor treating waste water from an anaerobic digestion process (Frison et al*.* 2015b, 2015a). In our study (Grey et al., 2022), NO_2_^−^ concentration was lowest in zone H (0.09 ppm), contrasting with zone M (0.21 ppm) where NO_2_^−^ was highest and PHA accumulation was lower, suggesting a role for NO_2_^−^. Inversely NH_4_^+^ and amino acid uptake in bacteria are significant metabolites for nitrogen assimilation (Hoch and Kirchman 1995; Patriarca and Iaccarino 2002; Böhm and Boos 2004), and in certain cases as stimulatory supplement for PHA production using long chain FAs (Lee et al. 1995; Benesova et al. 2017). The selection of N source by bacteria may also be an evolutionary adaption to resource competition across microbial communities (Treseder et al. 2011). Additionally, these adaptations are likely in response to vegetation N uptake with some plant species capable of dynamically adapting to assimilating different organic and inorganic N forms in response to ambient CO_2_ concentrations (Cott et al. 2018; Cott et al. 2020). PHA metabolism is a known adaptive physiological trait expressed by bacteria in response to nitrogen limitation (McKinley et al. 2005; La Rosa et al. 2014). In BI’s sediments, TOC: NH_4_^+^ was highest in zone H suggesting some N limitation despite this zone having the lowest value for TOC: N. The higher microbial biomass in the rhizospheric horizons of zone H sediments would indicate a higher demand for labile N sources.

The ability of bacteria to uptake medium and long chain length FAs in a controlled manner and polymerise into PHA chains, minimises the chances of cytoplasmic dissociation and aids in controlling intracellular pH, while also providing a reservoir for reducing equivalents (López et al. 2015; Prieto et al. 2016). This mechanism could also be extended as a service to surrounding microbial communities incapable of such adaptations by reducing sediment concentrations of FFAs. Organic acids have been recently shown as an important factor in P transformations and microbial availability in sediment (Menezes-Blackburn et al. 2016).This is an interesting prospect worthy of exploration and may also play a role in detoxification of localised FA accumulation in the rhizosphere, which is a known occurrence where plants produce and accumulate excessive FA’s under hyper salinity (Ivanova et al. 2009; Tsydendambaev et al. 2013). Indeed, recent publications have demonstrated the presence of PHA as a benefiting factor in symbiosis between prokaryotes and plants (Alves et al. 2019; Sun et al. 2019) where Sun et al. demonstrated contrasting symbiotic performances between wild strain and mutants lacking PHB synthases and depolymerases were revealed. Fatty acids and phenolic compounds have been previously mentioned as microbial inhibitors by Obruca et al. (Obruca, Sedlacek and Koller, 2021) during bacterial PHA production where lignocellulose hydrolysates are used as substrate. The authors highlighted potential metabolic disruption from the perspective of lowering intracellular pH, denaturing proteins and causing damage of membrane integrity. The high mineral contents in BI’s sediments provides a retentive matrix not only for toxic materials, but also assisting in the accrual of microbial necromass through formation of organo-mineral complexes (Creamer et al. 2019; Sokol and Bradford 2019).

In the context of higher mud content in vegetated sediments, a significant effect of charged particles is the ion exchange capacity of negatively charged clays. PAHs are also susceptible to silt/clay adsorption where various PAH molecules can form cationic dimers with intermediate binding energies between normal van der Waals complexes and chemical bonds (Qu et al. 2008). The surface sorption of aromatic compounds has been shown to be increasingly prevalent under higher salinity (Wang et al. 2010; Weston et al. 2010) and lower pH (Nanuam et al. 2013). Therefore, high Cl– and lower pH in marsh zones may fluctuate under tidal inundation, facilitating the release of more toxic organic and inorganic compounds at localised scales under such dynamic conditions as found in VCEs. This may be an additional significant trigger for PHA accumulation in some species of bacteria as previous studies have clearly shown the enhanced survival of PHA accumulating bacteria subjected to oxidative metal species (Obruca et al. 2016; Obruca et al. 2018)**.** The relatively high PHA µg/mg OC in zone H samples SM1, SM2 and SM 5 suggests that edaphic factors other than elevated OC content relate to community accumulation of PHA (Rothermich et al. 2000).

In this study PHA accumulation was strongly and positively related to OM constituents but also, with Pb, pH, high PAH pollution status and interestingly the presence of different microbial groups such as Fungi and Actinomycetes. As C increased there was a clear increase in bacteria such as Actinobacteria and Chloroflexi. These are prominent in degradation of more complex C substrates such as cellulose and show prevalence in chemically challenging environments including sewerage and wastewater treatment processes (Kragelund et al. 2007; Zhang et al. 2017).

In blue carbon sediments, long term anoxia and rapid burial are fundamental aspects of C sequestration, mainly through inhibition of bacteria mineralisation (Trevathan-Tackett et al. 2017). Further inhibition can occur through increased soil toxicity which is also inhibitory to optimum vegetation life-cycles. It is important for balance between inhibition of C mineralisation by bacteria and continued functioning of niche community members to sustain the holistic functioning of blue carbon habitats. The ability of bacteria to adapt to fluctuating and adverse geochemical changes using systematic lipid alterations presents such a dynamic mechanism. The presence of elevated metabolism disruptors in salt marsh zone sediments such as Pb, PAH, high salinity and excessive FA concentration may exert inhibiting conditions on certain bacteria, therefore reducing their respective abundances. The results in this study showed bacterial expression of metabolic stress to increase in response to elevated C, PAH and metals through alteration of lipid membrane, and accumulation of PHA. The presence of vegetation, elevated C and fluvial deposits coincided with a diversity in monomer composition, which likely reflects the increased uptake and regulation of longer chain FAs**.** However, more multidisciplinary research is needed to assess and decouple the microbial interactions dictating PHA cycling from the perspective of OM cycling dynamics, antagonistic competition and anthropogenic factors in different ecological contexts. This is especially important in the context of blue carbon sediments receiving anthropogenically derived allochthonous materials including sewage, attached microbes, pollutants and C substrate.

Coastal wetlands are highly important blue carbon habitats contributing to global C sequestration. Vegetation and associated microbiology are two fundamentally vital drivers of efficient capture, processing and storage of C in blue carbon zones. As climate change and human population advance under current projections, so too will the C chemistry of blue carbon sediments with increasing C mineralisation (Dieleman et al. 2016) and anthropogenic inputs. The multifunctional PHA metabolic system provides an ecological advantage to bacteria and potentially other cohabiting organisms under a multitude of stresses, many of which have been identified in coastal wetland sediments. PHA is a transient C storage system, however, this ‘power-pack’ storage system maintains the presence of valuable biological players in C cycling.

## Supplementary Information

Below is the link to the electronic supplementary material.

Supplementary file1 (DOCX 7901 kb)

## Data Availability

Enquiries about data availability should be directed to the authors.
