# Supplementary material for: Biogeochemical properties of blue carbon sediments influence the distribution and monomer composition of bacterial polyhydroxyalkanoates (PHA)
Source: Biogeochemistry. 2023 Jan 10;162(3):359–80. doi: 10.1007/s10533-022-01008-5 (PMC9971093; doi:10.1007/s10533-022-01008-5)
Supplement: Supplementary file 1 — Supplementary file1 (DOCX 7901 kb) [file 10533_2022_1008_MOESM1_ESM.docx]

**Supplementary information**

**Materials and methods**

**Lipid biomarker analysis**

**Lipid extraction**

Freeze-dried sediment aliquots of 5g were extracted using a modified Bligh and Dyer extraction ^13–15^. A final extraction step was performed prior to solvent ratio adjustment with the addition of 15ml of chloroform, placement of the tubes in a water bath at 40 °C for 30 mins and shaking for 1hr at 200 rpm This additional step was carried out to maximise the dissolution of the non-polar PHA granules released from lysed bacterial cells in a notably heterogeneous matrix. After centrifuging, the supernatant was combined with previous extracts in respective separating funnels. The solvent ratio was adjusted to 1:1:0.9, the extracts were left for 14 hours to allow for diphasic separation. The bottom organic layer was collected in a round bottom flask after filtration through GF/a filter papers containing anhydrous sodium sulphate and the aqueous/interphase layers were washed through with 5 ml aliquots of chloroform and collected in respective flasks. Flasks were de-aired using Nitrogen (N_2_) gas and sealed with glass stoppers. The total lipid extracts were condensed by rotary evaporation, transferred to vials with activated copper and de-aired using Nitrogen (N_2_) gas before shaking for 12 hrs to remove sulfur which causes chromatographic interferences ^16^.

**Lipid fractionation**

Lipids were fractionated using solid phase extraction (SPE) on *Agilent Bond Elute NH_2_* columns (aminopropyl solid phase -500 g 3 ml) as described by Pinkart et al ^17^. A deviation from the Pinkart method involved the combination of CHCl_3_ and acetone eluted fractions during SPE and consequent derivatisations for analysis of PHAs, due to the higher solubility of PHAs in chloroform. This approach has been previously utilised in a study of PHAs in a marine environment ^18^.

**PLFA derivatisation and GCMS analysis**

PLFAs were derivatised to produce fatty acid methyl esters (FAMEs) utilising sodium methoxide and monosaturated bonds were identified in subsequent FAMEs by DMDS substitution with all lipids analysed by GCMS as described in the following references ^19,20^. The data was processed using Chemstation software, combining mass spectral library databases (NIST and Wiley), spectra interpretation, retention times, specific ion extracted chromatograms and referenced literature to confirm presence of identified compounds ^19,20^. Lipids were quantified relative to an external calibration curve using methyl tetradecanoate and cholestane was used as an internal standard.

**PHA derivatisation and GC-MS analysis**

The chloroform/acetone fraction collected for PHA analysis was dried down under a stream of nitrogen. The remaining extract with the polymer dried to the walls of the vial was carefully washed with 3 x1 ml aliquots of ethanol , followed by 3 x1 ml aliquots of diethyl ether to remove excessive neutral lipid and free fatty acid compounds likely to cause chromatographic interference and derivatised as described by Findlay et al ^21^. A new GC method was developed for PHA analysis as adapted from Findlay and White ^21,22^ is described in supplementary information section 2.

**GCMS analysis of PHA monomer derivatives**

The column was a fused silica capillary column (30 m × 0.25 mm i.d.) with a film thickness of 0.25 μm (HP-5MS, Agilent). Ultra-high purity helium (BIP-X47Sgrade, Air Products) was used as the carrier gas with a flow rate of 1 mL min−1. The sample (1μl) was injected with a 2:1 split ratio. The GC inlet port temperature was set at 250⁰C. An initial oven temperature of 60⁰C was held for 1 min, followed by a ramp of 5⁰C /min to 280⁰C, held for 1min and finally ramped at 25⁰C /min to 310⁰C for a 20min hold time. The total run was 66mins for this PHA method. The data was processed using Chemstation software. A series of custom made 3OHA standards (bacteria source) were obtained from *BIOPLASTECH* (Dr. Kevin O’Connor, University College Dublin) and used to generate a standard curve. The mix contained 3-hydroxy butanoic acid (3OHB) , 3-hydroxy-valeric acid (3OHV), 3-hydroxy hexanoic acid (3OHH), 3-hydroxy heptanoic acid (3OHHP), 3-hydroxy octanoic acid (3OHO), 3-hydroxy-decanoic acid (3OHD) and 3-hydroxy-dodecanoic acid (3HDoD). The 3-hydroxyacids were subjected to the same derivatization as per sediment extract. Individual compounds were identified combining mass spectral library databases (NIST and Wiley), standard spectra interpretation, retention times, specific ion extracted chromatograms and published literature. In previous work (not reported here), a pure culture of pseudomonas (obtained from ATCC®) was grown under aseptic conditions using mono-unsaturated fatty acid substrates to facilitate synthesis of mono-unsaturated PHA monomers. The analysis of the isolated unsaturated monomers by GC-MS provided spectra to enhance identification of unknown monomers otherwise unavailable from online databases. Where no standards or examples of spectra were available for LCL monomers and unsaturated monomers, mass spectra fragmentation patterns and respective diagnostic ions were studied from known compounds to estimate RT and thus predicted fragments for potential monomers e.g. Ion m/z 117 representing bond cleavage at the β carbon and molecular ion mass (where possible) to identify addition of a carbon as the chain length of monomers increases.


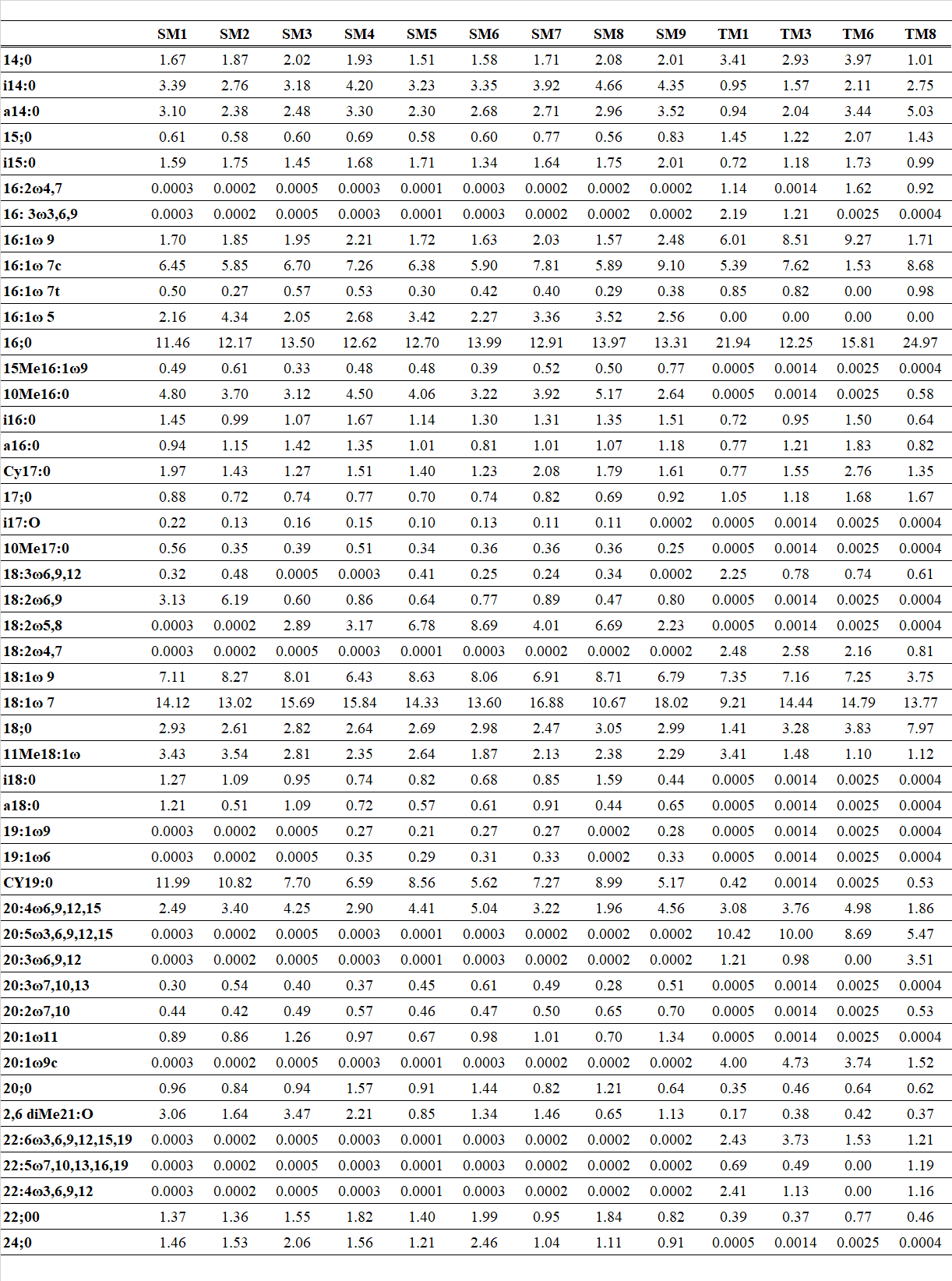
Table SI.1: Mean concentrations for detected PLFAs (ug/g dws ) at individual sample sites

Table SI.2: Mean concentration (µg /g dws) of individual PHA monomers quantified to generate total PHA µg g-1 across each sample site. Samples were analysed in the saltmarsh and tidal mud zones

|  | Salt Marsh | | | | | | | | | Tidal Mud | | | |
| --- | --- | --- | --- | --- | --- | --- | --- | --- | --- | --- | --- | --- | --- |
| PHA monomer µg /g:  3-Hydroxy -C (n) | SM1 | SM2 | SM3 | SM4 | SM5 | SM6 | SM7 | SM8 | SM9 | TM1 | TM3 | TM6 | TM8 |
| 3OH-4:0 | 17.85 | 42.15 | 2.34 | 11.83 | 33.94 | 10.19 | 1.28 | 8.82 | 2.65 | 1.45 | 0.04 | 0.12 | 1.15 |
| 3Me3OH-4:0 | 0.58 | 2.42 | 0.49 | - | - | - | - | - | - | 5.20 | 0.28 | 0.62 | 0.92 |
| 3OH-5:0 | 71.59 | 64.70 | 12.03 | 30.92 | 39.83 | 18.44 | 16.04 | 15.08 | 10.86 | - | - | - | - |
| 2Me4OHV | 0.79 | 0.67 | 0.22 | - | - | - | - | - | - | - | - | - | - |
| 3OH-6:0 | 0.43 | 0.18 | 0.07 | 0.23 | 0.10 | 0.09 | - | - | - | - | - | - | - |
| 3OH-7:0 | 0.68 | 1.70 | 0.06 | 0.06 | 0.33 | - | 0.04 | 0.31 | - | - | - | - | - |
| 3OH-8:0 | 1.06 | 0.45 | 0.36 | 0.14 | 0.27 | 0.22 | 0.18 | 0.07 | 0.04 | - | - | - | - |
| 3OH-9:0 | 0.09 | 0.11 | 0.12 | - | - | - | - | - | - | - | - | - | - |
| 3OH-10 | 3.53 | 1.10 | 0.52 | 0.21 | 0.46 | 0.29 | 0.36 | 0.36 | 0.12 | - | - | - | - |
| 3OH12:1w | 0.60 | 0.29 | 0.08 | - | - | - | - | - | - | - | - | - | - |
| 3HO12:O | 4.07 | 1.71 | 0.45 | 0.21 | 0.38 | 0.25 | 0.24 | 0.11 | 0.13 | - | - | - | - |
| 3OH13:O | 4.73 | 3.12 | 0.87 | 0.49 | 0.88 | 0.50 | 0.45 | 0.22 | 0.19 | - | - | - | - |
| 3OH14:1w | 5.16 | 4.34 | 1.42 | 0.87 | 1.59 | 0.91 | 0.72 | 0.40 | 0.31 | - | - | - | - |
| 3OH14:O | 3.03 | 1.85 | 0.58 | 0.96 | 1.45 | 0.92 | 0.45 | 0.31 | 0.31 | - | - | - | - |
| 3OH16:1w | 1.80 | 2.80 | 1.11 | 0.78 | 1.56 | 0.81 | 1.30 | 0.47 | 0.54 | - | - | - | - |
| 3OH18:1w | 6.71 | 4.13 | 0.85 | - | 0.80 | 0.39 | 1.11 | 0.41 | 0.51 | - | - | - | - |
| Total PHA ug g soil | 122.68 | 131.71 | 21.57 | 46.69 | 81.59 | 32.99 | 22.16 | 26.55 | 15.67 | 6.64 | 0.32 | 0.74 | 2.07 |

Figure SI.1: Boxplots show results of significantly different microbial PLFA indices (n=39) between zones H, M and T after Kruskal-Wallis tests, where significance was set at level, alpha (p) = 0.05. Significant differences between H vs M, and M vs T zones is denoted by ‘ ’ and ‘Δ’ respectively, above the relevant boxplots .


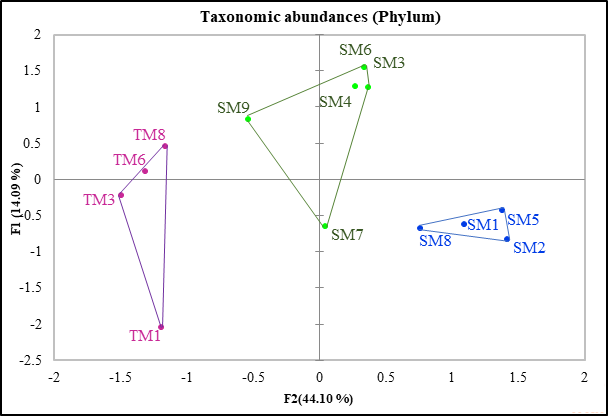

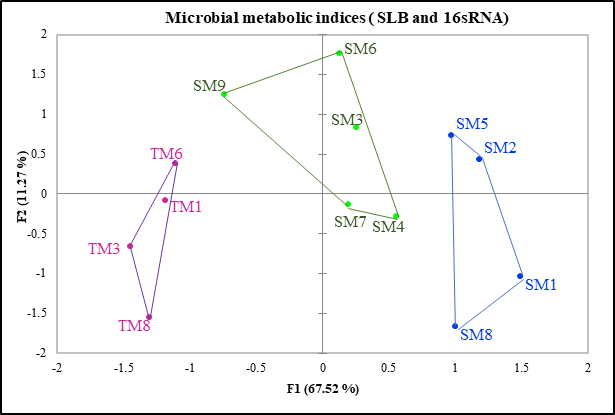

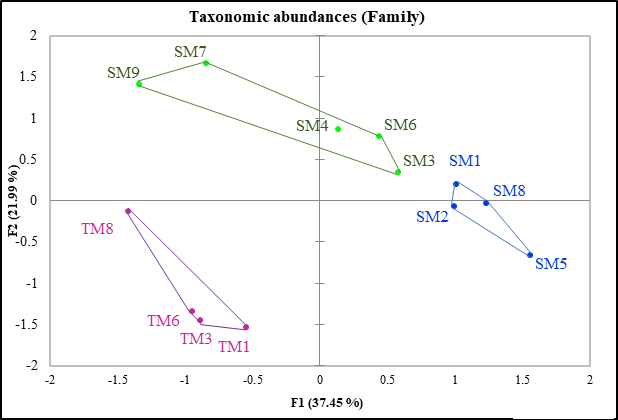

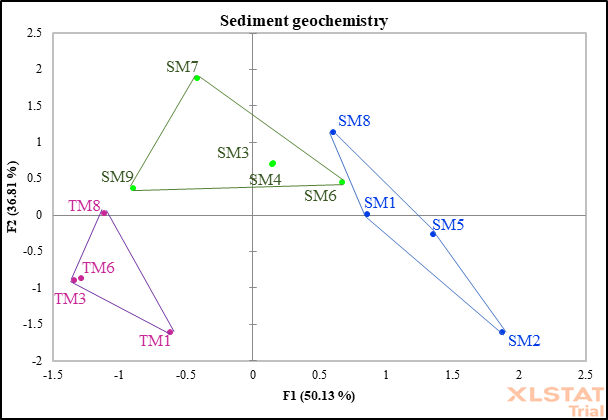


Figure SI.2: In PCA of geochemical variables, taxonomic composition (16s rRNA for phylum and family), and biomarker analysis , the sample sites showed separation into groups representing upper marsh (H), mid-lower marsh (M) and tidal flats (T) zones, revealing similar patterns between samples in multivariate space (figure…). Points are coloured by soil horizon and the convex hulls are drawn and highlighted for each horizon groupings Geochemical variables for PCA were chosen after examining covariation results and representative variables were selected (pH, %OM, NO2-, PAH, TP, clay, EC) for OM fractions, metals, inorganic particles, anthropogenic influences, nutrients and sediment chemistry, while minimizing intercorrelations where possible. There was a clear and consistent separation of sample sites along the first principal component axis (F1) in all cases with highest total % variance explained for combined axis (F1 and F2) in order of sediment geochemistry (86.94%), microbial metabolic indices (78.79%) and taxonomic composition for family (59.34%) and phylum (58.19%). In order to identify the geochemical properties and taxa at a phylum level which contribute most to the separation between sediment zones, the non-parametric Kruskal-Wallace H test for differences between groups was applied (Kruskal and Wallis, 1952).

Table SI.3: Table displaying the mean and std deviation of the top 24 most abundant phyla across grouped sites representing gradient zones.


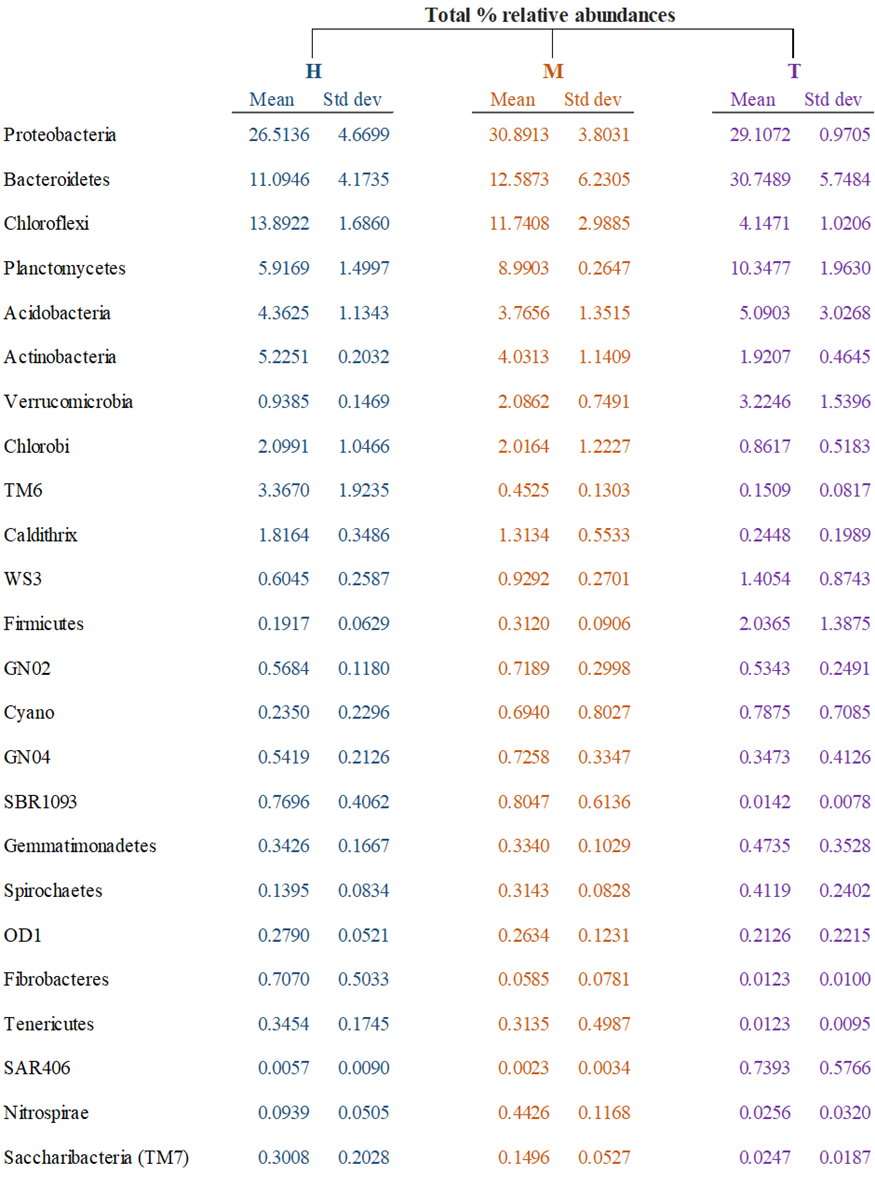


Figure SI.4.2: Principal co-ordinate analysis of individual sample sites from zones H, M and T using bacteria operational taxonomic units (OTU).


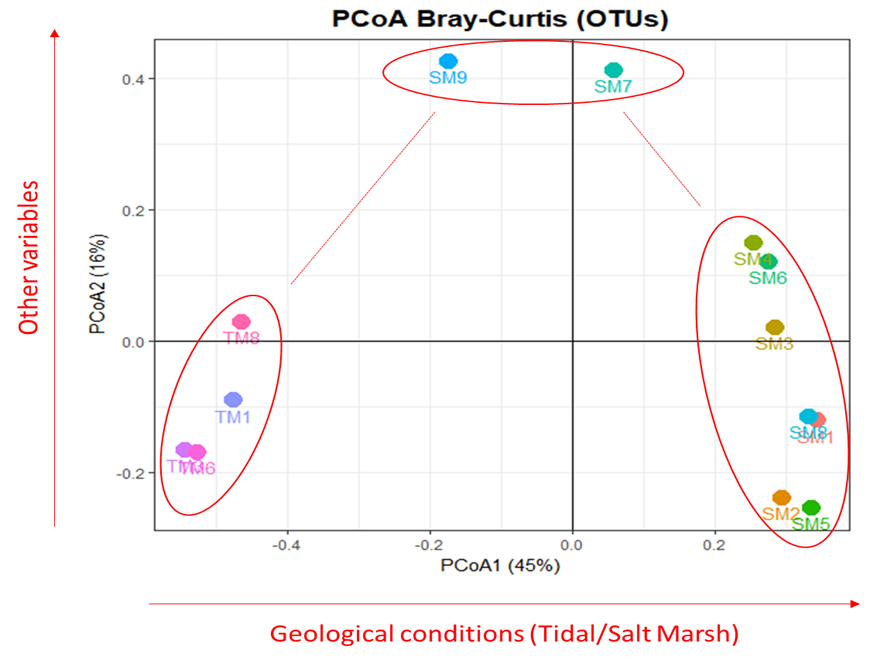


Figure SI.3: Principal co-ordinate analysis of individual sample sites from zones H, M and T using bacteria operational taxonomic units (OTU).

**4.3 Metagenomics results**

Family classification introduced further distribution diversities for all zones ( Table 2). Other α-Proteobacteria more abundant across marsh zones were families Rhodosprillaceae and Hyphomicrobiaceae, both containing metabolically diverse members involved in nitrification, denitrification , sulfur oxidation and hydrocarbon degradation processes, including both photoheterotrophs and chemoheterotrophs with tolerance for low oxygen conditions ^47,48^. Rhodobacteracae (α- Proteobacteria) is a known marine dwelling family present in significant abundance in zone T alongside other Proteobacteria families Marinicellaceae (Ɣ – gamma), OM60 (Ɣ), Desulfobulbaceae (δ - delta) and Helicobacteraceae (ɛ-Epsilon). The Beta proteobacteria (β) were the most underrepresented in the phylum, however, the ammonia oxidizing family – Nitrosomandaceae – were the main contributors to this group, present exclusively in the marsh zones, with significantly elevated levels in the mid marsh regions (M=0.17%, p<0.05) ^49^. The phylum Bacteroidetes, flourished in the T zone with 30.75% abundance, showing significantly higher abundance than in both sampled marsh zones (p<0.05, M=12.59%, H=11.09%). The predominant Bacteroidetes family identified were Flavobacteriaceae across all zones but highest in zone T, whereas family group, Flammeovirgaceae increased on the gradient up through the marsh zones (p>0.05). Plantomycetes present a reversing trend to Chloroflexi with the highest abundance in zone T (10.35%), decreasing through zone M (8.99%) and significantly lowest in H (5.92%, p<0.05) than the preceding zones. Many sub divisions within the Plantomycetes phylum play an important role in anaerobic oxidation of ammonium in a diversity of sediments, soils, microbial mats and in environments such as transitional water bodies ^50–52^. Firmicutes (2.04%) and SAR406 (0.74%) were highest in this zone, indicating a possible marine niche as abundance decreased significantly up through the gradient towards the upper marsh. In marine mudflats, planktonic debris provides polysaccharide and proteinaceous substrate for aerobic Flavobacteriaceae and facultative anaerobe phyla, Spirochetes and Plantomycetes ^53–55^. The latter is a diverse domain with many subdivisions providing niche roles in symbiotic nitrogen and sulfur cycling through many redox zones, integral to microbial mat communities in marine settings, evident by the highest presence of cyanobacteria in zone T ^56^ Sulfate reducing Deltaproteobacteria families Desulfobacteraceae and Desulfobulbaceae, strict anaerobes, likely represent the lower horizons of the 10 cm sample depth. The gram positive phylum Firmicutes shares this anaerobic fermentive type metabolism with highest relative abundance in zone T, coinciding with a significantly higher pH which has been shown to mobilise dissolved organic matter (DOM) in sediments and have a stimulatory effect on elevating firmicutes abundance ^57^. The consortium of fermentive phyla present is interesting in the context of redox conditions and the PHA monomer composition.

In zone M , Nitrospirae were significantly highest (0.45%) than each of zones T (0.03%) and H (0.09). Actinobacteria a gram +, heterotrophic and an entophytic symbiont was significantly highest (p<0.05) in the upper reaches of the marsh, zone H (H=5.23%, M=4.03%, and T=1.92%) ^58^. Also highest in Zone H were pathogenic candidate phylum TM6S (3.37%, p<0.05), host dependent and associated with anaerobic conditions ^59,60^. Fibrobacteres an important phylum of heterotrophs associated with cellulose degradation were low in relative abundance overall, however, this phyla presented a significantly higher representation in zone H (0.71%, p<0.05) ^61,62^.

Some bacteria showed similar abundance between zones with Tenericutes, SBR1093, Saccharibacteria (TM7) and Chlorobi, with sulfur oxidising members showing a significant preference for marsh sediments. Sulfur oxidation contributes to lowering of pH and mobilisation of heavy metals in sediments, and many members are present in Beta- and Gamma proteobacteria classes ^63^. Zone M also showed significant commonalities with zone T, where faculatively anaerobic, sulfate reducing phyla Verrumicrobia were present in similar abundances ^64,65^. Additionally, two phyla with robust metabolisms suited to dynamic lifestyles in microbial mat debris, Spirochetes and Plantomycetes, had similar mean abundances for both zones T and M, with significant decreases as the marsh gradient reaches zone H ^55,66^. A mixture of phyla and candidates, Proteobacteria, Acidobacteria, Cyanobacteria, WS1, GN02, GN04, Gemmatimonadetes, and OD1, all colonise successfully throughout the gradient zones with no significant group-wise response to changing edaphic factors. This is unsurprising at the broader taxonomic classification of phylum. Bacteria can contain a multitude of subdivisions at refined taxa levels (i.e. class, family, genus and species). Ultimately possessing both general and niche roles in sediments through many redox gradients, plant-host interactions, carbon/nutrient variations, anthropogenic stresses and antagonist biotic factors, all of which are represented in vegetated coastal ecosystems ^67^. Filamentous, monoderm phylum, Chloroflexi were represented highly in H and M zones at 13.89% and 11.74% respectively, each zone having significantly higher values (p<0.05) in contrast to zone T with a decrease down the gradient to 4.15%.Chloroflexi predominance in the marsh zones was characterised by the high abundance of sub-phylum Anaerolineae and families A4b, SJA-101 and UC-DRC31. Common traits including filamentous morphology as well as non-motile, non-sporulation, and gram-negative characteristics, with known roles in cellulolytic degradation and fermentative traits during wastewater treatment and anaerobic digestion processes ^68–71^. The response of bacteria to a changing environment is a function of its potential genetic expressions, but importantly includes the ability to network with other biological players (e.g. Bacteria, Fungi, Archaea, micro invertebrates and macrophytes) across many mutualistic and parasitic interactions.

Table SI.4: results for OTU richness and evenness at individual sample sites across Bull island.


Figure SI.4: Boxplots of distributions for OTU richness and evenness between groups H, M and T. Both metrics had highest values in zone M to a significance level at p<0.05.

Figure SI.5: CCA depicting the relationships between representative geochemical variables, microbial community composition and stress status (using 16srRNA bacteria phylum, lipid biomarker taxonomic classes and metabolic status indices). Graph shows site distributions under the constraints of sediment geochemical variables – % Mud (clay and silt), PAH (µg g), NO_2_^-^ and EC (ms/cm).

Figure SI.6: Interpretation of PHA and PLFA distributions at individual sites: A) total PHA and PLFA per µg/g dw sediment, B) total PHA and PLFA normalised to mg OC, C) PHA: PLFA ratios at each site, D) sum of SCL, MCL and LCL monomers at each sites.


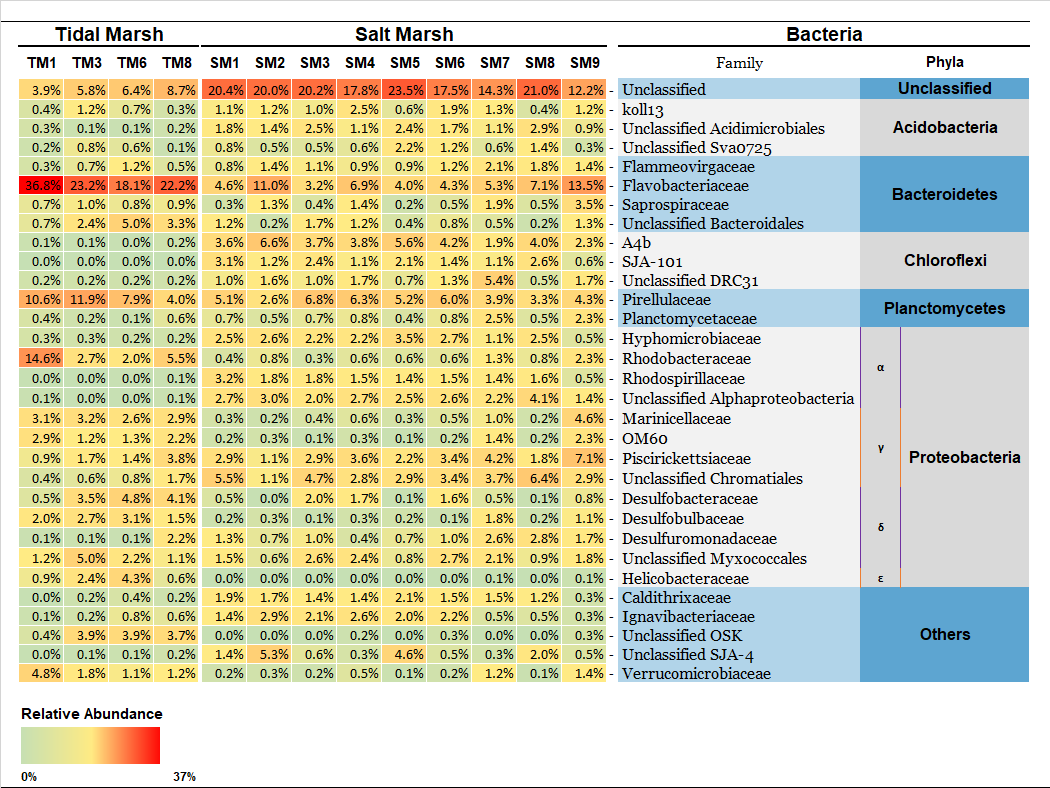


Figure SI.7 : Quantitative taxonomic analysis to family level. Presented are the top 30 abundant bacteria family groups

1. **Conceptual diagram**

The conceptual diagram ( Figure 7) was generated using data from statistical analysis. Firstly, group or zone ranges for respective geochemical parameters were attained from descriptive statistics results. PHA monomers were assigned to zones based on qualitative data from GCMS results. The results from lipid biomarker and 16s rRNA metagenomics analysis were statistically tested as described above. Bacterial phyla and microbial groups were assigned to zones under respective diagram heading where testing revealed a significant (p<0.05) presence. Projected zones are conceptual and occurred where microbial groups were not significantly different between two zones, but presented significantly lower in the third zone e.g. Chloroflexi were significantly higher in zones H and M when compared to zone T, however the results for Chloroflexi were not statistically different between H and M, thereafter named zone H-M. Any phyla not statistically different between all zones were deemed common across the transect gradient.

**References**

1. Healy, B. Fauna of the Salt-Marsh , North Bull Island , Dublin. *Proc. R. Ir. Acad. B.* **75**, 225–244 (1975).

2. Simpson, S. L. *et al.* Climate-driven mobilisation of acid and metals from acid sulfate soils. *Mar. Freshw. Res.* **61**, 129–138 (2010).

3. Gross, C. D. & Harrison, R. B. The Case for Digging Deeper: Soil Organic Carbon Storage, Dynamics, and Controls in Our Changing World. *Soil Syst.* **3**, 28 (2019).

4. Hoogsteen, M. J. J., Lantinga, E. A., Bakker, E. J., Groot, J. C. J. & Tittonell, P. A. Estimating soil organic carbon through loss on ignition: Effects of ignition conditions and structural water loss. *Eur. J. Soil Sci.* **66**, 320–328 (2015).

5. Rayment, G. E. & Higginson, F. R. Soil survey standard test method for electrical conductivity. *Aust. Lab. Handb. Soil Water Chem. Methods, Melbourne, Inkata Press. (Australian Soil L. Surv. Handbooks)* **3**, 1–10 (1992).

6. Radu, T. & Diamond, D. Comparison of soil pollution concentrations determined using AAS and portable XRF techniques. *J. Hazard. Mater.* **171**, 1168–1171 (2009).

7. Radu, T. *et al.* Portable X-Ray Fluorescence as a Rapid Technique for Surveying Elemental Distributions in Soil. *Spectrosc. Lett.* **46**, 516–526 (2013).

8. IAEA. *Sampling, storage and sample prepararation for X ray fluorescence analysis of environmental materials*. *IAEA-Tecdoc 950* vol. 28 (1997).

9. Vaasma, T. Grain-size analysis of lacustrine sediments: A comparison of pre-treatment methods. *Est. J. Ecol.* **57**, 231–243 (2008).

10. Verardo, D. J., Froelich, P. N. & McIntyre, A. Determination of organic carbon and nitrogen in marine sediments using the Carlo Erba NA-1500 analyzer. *Deep Sea Res. Part A. Oceanogr. Res. Pap.* **37**, 157–165 (1990).

11. Cogan, D. *et al.* The development of an autonomous sensing platform for the monitoring of ammonia in water using a simplified Berthelot method. *Anal. Methods* **6**, 7606–7614 (2014).

12. Ivanov, K. *et al.* ICP determination of phosphorous in soils and plants. *19th World Congr. Soil Sci. Soil Solut. a Chang. World* 71–74 (2010).

13. Bligh, E. G. & Dyer, W. J. A RAPID METHOD OF TOTAL LIPID EXTRACTION AND PURIFICATION. *Can. J. Biochem. Physiol.* **37**, 911–917 (1959).

14. Fang, J. & Findlay, R. H. The use of a classic lipid extraction method for simultaneous recovery of organic pollutants and microbial lipids from sediments. *J. Microbiol. Methods* **27**, 63–71 (1996).

15. White, D. C., Ringelberg, D. B., Macnaughton, S. J., Alugupalli, S. & Schram, D. White 1996 review of pha and signature lipid biomarker analysis for quantitative assessment of in situ environmental microbial ecology.pdf. *Mol. Markers Environ. Geochemistry* **671**, 22–34 (1997).

16. Canton, L. & Grimalt, J. O. Gas-chromatographic-mass spectrometric characterization of polycyclic aromatic mixtures in polluted coastal sediments. *J. Chromatogr.* **607**, 279–286 (1992).

17. Pinkart, H. C., Devereux, R. & Chapman, P. J. Rapid separation of microbial lipids using solid phase extraction columns. *J. Microbiol. Methods* **34**, 9–15 (1998).

18. Guezennec, J. *et al.* Occurrence of 3-hydroxyalkanoic acids in sediments from the Guaymas basin (Gulf of California). *FEMS Microbiol. Ecol.* **26**, 335–344 (1998).

19. O’Reilly, S. S. *et al.* Shallow water methane-derived authigenic carbonate mounds at the Codling Fault Zone, western Irish Sea. *Mar. Geol.* **357**, 139–150 (2014).

20. Murphy, B. T. *et al.* The occurrence of PAHs and faecal sterols in Dublin Bay and their influence on sedimentary microbial communities. *Mar. Pollut. Bull.* **106**, 215–224 (2016).

21. Findlay, R. H. & White, D. C. Polymeric Beta-Hydroxyalkanoates from Environmental Samples and Bacillus megaterium. *Appl. Environ. Microbiol.* **45**, 71–78 (1983).

22. Tan, G.-Y. A. *et al.* Enhanced gas chromatography-mass spectrometry method for bacterial polyhydroxyalkanoates analysis. *J. Biosci. Bioeng.* **117**, 379–382 (2014).

23. Frostegard, A. *et al.* Shifts in the structure of soil microbial communities in limed forests as revealed by phospholipid fatty acid analysis. *Soil Biol. Biochem.* **25**, 723–730 (1993).

24. Bossio, D. A. *et al.* Soil microbial community response to land use change in an agricultural landscape of western Kenya. *Microb. Ecol.* **49**, 50–62 (2005).

25. Willers, C., Jansen van Rensburg, P. J. & Claassens, S. Phospholipid fatty acid profiling of microbial communities-a review of interpretations and recent applications. *J. Appl. Microbiol.* **119**, 1207–1218 (2015).

26. Rütters, H., Sass, H., Cypionka, H. & Rullkötter, J. Phospholipid analysis as a tool to study complex microbial communities in marine sediments. *J. Microbiol. Methods* **48**, 149–160 (2002).

27. Vestal, J. R. & White, D. C. Lipid Analysis in Microbial Ecology: Quantitative approaches to the study of microbial communities. *Bioscience* **39**, 535–541 (1989).

28. Chaudhary, D. R., Kim, J. & Kang, H. Influences of Different Halophyte Vegetation on Soil Microbial Community at Temperate Salt Marsh. *Microb. Ecol.* **75**, 729–738 (2018).

29. Olsson, P. A., Bååth, E., Jakobsen, I. & Söderström, B. The use of phospholipid and neutral lipid fatty acids to estimate biomass of arbuscular mycorrhizal fungi in soil. *Mycol. Res.* **99**, 623–629 (1995).

30. Olsson, P. A. Signature fatty acids provide tools for determination of the distribution and interaction of mycorrhizal fungi. *FEMS Microbiol. Ecol.* **29**, 303–310 (1999).

31. Bååth, E. & Anderson, T. H. Comparison of soil fungal/bacterial ratios in a pH gradient using physiological and PLFA-based techniques. *Soil Biol. Biochem.* **35**, 955–963 (2003).

32. Fierer, N., Schimel, J. P. & Holden, P. A. Variations in microbial community composition through two soil depth profiles. *Soil Biol. Biochem.* **35**, 167–176 (2003).

33. Allison, V. J., Yermakov, Z., Miller, R. M., Jastrow, J. D. & Matamala, R. Using landscape and depth gradients to decouple the impact of correlated environmental variables on soil microbial community composition. *Soil Biol. Biochem.* **39**, 505–516 (2007).

34. Grogan, D. W. & Cronan, J. E. Cyclopropane ring formation in membrane lipids of bacteria. *Microbiol. Mol. Biol. Rev.* **61**, 429–41 (1997).

35. Findlay, R. H. & White, D. C. Polymeric beta-hydroxyalkanoates from environmental samples and Bacillus megaterium. *Appl. Environ. Microbiol.* **45**, 71–78 (1983).

36. Green, C. T. & Scow, K. M. Analysis of phospholipid fatty acids (PLFA) to characterize microbial communities in aquifers. *Hydrogeol. J.* **8**, 126–141 (2000).

37. Foster, L. J. R., Saufi, A. & Holden, P. J. Environmental concentrations of polyhydroxyalkanoates and their potential as bioindicators of pollution. *Biotechnol. Lett.* **23**, 893–898 (2001).

38. McKinley, V. L., Peacock, A. D. & White, D. C. Microbial community PLFA and PHB responses to ecosystem restoration in tallgrass prairie soils. *Soil Biol. Biochem.* **37**, 1946–1958 (2005).

39. Rothermich, M. M., Guerrero, R., Lenz, R. W. & Goodwin, S. Characterization, seasonal occurrence, and diel fluctuation of poly(hydroxyalkanoate) in photosynthetic microbial mats. *Appl. Environ. Microbiol.* **66**, 4279–4291 (2000).

40. Spivak, A. C., Sanderman, J., Bowen, J. L., Canuel, E. A. & Hopkinson, C. S. Global-change controls on soil-carbon accumulation and loss in coastal vegetated ecosystems. *Nat. Geosci.* **12**, 685–692 (2019).

41. Soergel, D. A. W., Dey, N., Knight, R. & Brenner, S. E. Selection of primers for optimal taxonomic classification of environmental 16S rRNA gene sequences. *ISME J.* **6**, 1440–1444 (2012).

42. Caporaso, J. G. et al. QIIME allows analysis of high- throughput community sequencing data. *Nature Publishing Group* vol. 7 335–336 (2010).

43. Edgar, R. C. Search and clustering orders of magnitude faster than BLAST. *Bioinformatics* **26**, 2460–2461 (2010).

44. DeSantis, T. Z. *et al.* Greengenes, a chimera-checked 16S rRNA gene database and workbench compatible with ARB. *Appl. Environ. Microbiol.* **72**, 5069–5072 (2006).

45. Oksanen, A. J. *et al.* Package ‘ vegan ’. (2018).

46. DT, O., AA, A. & OE, O. Heavy Metal Concentrations in Plants and Soil along Heavy Traffic Roads in North Central Nigeria. *J. Environ. Anal. Toxicol.* **05**, 6–10 (2015).

47. Wang, K. *et al.* Regional variations in the diversity and predicted metabolic potential of benthic prokaryotes in coastal northern Zhejiang, East China Sea. *Sci. Rep.* **6**, 1–12 (2016).

48. Matturro, B., Viggi, C. C., Aulenta, F. & Rossetti, S. Cable bacteria and the bioelectrochemical Snorkel: The natural and engineered facets playing a role in hydrocarbons degradation in marine sediments. *Front. Microbiol.* **8**, 1–13 (2017).

49. Prosser, J., M.Head, I. & Y.Stein, L. The Family Nitrosomonadaceae. in *The Prokaryotes: Alphaproteobacteria and Betaproteobacteria* 901–918 (2013). doi:10.1007/978-3-642-30197-1.

50. Jetten, M. S. M. *et al.* Anaerobic ammonium oxidation by marine and freshwater planctomycete-like bacteria. *Appl. Microbiol. Biotechnol.* **63**, 107–114 (2003).

51. Sinninghe Damsté, J. S., Rijpstra, W. I. C., Geenevasen, J. A. J., Strous, M. & Jetten, M. S. M. Structural identification of ladderane and other membrane lipids of planctomycetes capable of anaerobic ammonium oxidation (anammox). *FEBS J.* **272**, 4270–4283 (2005).

52. Strous, M. *et al.* Missing lithotroph identified as new planctomycete. *Nature* **400**, 446–449 (1999).

53. Lage, O. M. & Bondoso, J. Planctomycetes and macroalgae, a striking association. *Front. Microbiol.* **5**, 1–9 (2014).

54. Wong, H. L., Smith, D. L., Visscher, P. T. & Burns, B. P. Niche differentiation of bacterial communities at a millimeter scale in Shark Bay microbial mats. *Sci. Rep.* **5**, 1–17 (2015).

55. Dong, X. *et al.* Fermentative Spirochaetes mediate necromass recycling in anoxic hydrocarbon-contaminated habitats. *ISME J.* **12**, 2039–2050 (2018).

56. Paerl, H. W., Pinckney, J. L. & Steppe, T. F. Cyanobacterial-bacterial mat consortia: Examining the functional unit of microbial survival and growth in extreme environments. *Environ. Microbiol.* **2**, 11–26 (2000).

57. Anderson, C. R. *et al.* Rapid increases in soil pH solubilise organic matter, dramatically increase denitrification potential and strongly stimulate microorganisms from the Firmicutes phylum. *PeerJ* **2018**, (2018).

58. Zhao, K. *et al.* Actinobacteria associated with Glycyrrhiza inflata Bat. are diverse and have plant growth promoting and antimicrobial activity. *Sci. Rep.* **8**, 1–13 (2018).

59. Solden, L., Lloyd, K. & Wrighton, K. The bright side of microbial dark matter: Lessons learned from the uncultivated majority. *Curr. Opin. Microbiol.* **31**, 217–226 (2016).

60. Yeoh, Y. K., Sekiguchi, Y., Parks, D. H. & Hugenholtz, P. Comparative genomics of candidate phylum tm6 suggests that parasitism is widespread and ancestral in this lineage. *Mol. Biol. Evol.* **33**, 915–927 (2016).

61. Ransom-Jones, E., Jones, D. L., McCarthy, A. J. & McDonald, J. E. The Fibrobacteres: An Important Phylum of Cellulose-Degrading Bacteria. *Microb. Ecol.* **63**, 267–281 (2012).

62. Rahman, N. A. *et al.* A phylogenomic analysis of the bacterial phylum fibrobacteres. *Front. Microbiol.* **6**, (2016).

63. Tourna, M., Maclean, P., Condron, L., O’Callaghan, M. & Wakelin, S. A. Links between sulphur oxidation and sulphur-oxidising bacteria abundance and diversity in soil microcosms based on soxB functional gene analysis. *FEMS Microbiol. Ecol.* **88**, 538–549 (2014).

64. Freitas, S. *et al.* Global distribution and diversity of marine Verrucomicrobia. *ISME J.* **6**, 1499–1505 (2012).

65. Cardman, Z. *et al.* Verrucomicrobia are candidates for polysaccharide-degrading bacterioplankton in an Arctic fjord of Svalbard. *Appl. Environ. Microbiol.* **80**, 3749–3756 (2014).

66. Stephens, E. A., Braissant, O. & Vissher, P. T. Spirochetes and salt marsh microbial mat geochemistry: Implications for the fossil record. *Carnets géologie (Notebooks Geol.* **09**, 1–11 (2008).

67. Dini-Andreote, F. *et al.* Dynamics of bacterial community succession in a salt marsh chronosequence: Evidences for temporal niche partitioning. *ISME J.* **8**, 1989–2001 (2014).

68. Kragelund, C. *et al.* Identity, abundance and ecophysiology of filamentous Chloroflexi species present in activated sludge treatment plants. *FEMS Microbiol. Ecol.* **59**, 671–682 (2007).

69. Björnsson, L., Hugenholtz, P., Tyson, G. W. & Blackall, L. L. Filamentous Chloroflexi (green non-sulfur bacteria) are abundant in wastewater treatment processes with biological nutrient removal c cThe EMBL accession numbers for the sequences reported in this paper are X84472 (strain SBR1029 16S rDNA), X84474 (strain. *Microbiology* **148**, 2309–2318 (2002).

70. Speirs, L. B. M., Rice, D. T. F., Petrovski, S. & Seviour, R. J. The Phylogeny, Biodiversity, and Ecology of the Chloroflexi in Activated Sludge. *Front. Microbiol.* **10**, (2019).

71. Szymańska, S. *et al.* Bacterial microbiome of root-associated endophytes of Salicornia europaea in correspondence to different levels of salinity. *Environ. Sci. Pollut. Res.* **25**, 25420–25431 (2018).
